# Genetic and physiological responses to light quality in a deep ocean ecotype of *Ostreococcus*, an ecologically important photosynthetic picoeukaryote

**DOI:** 10.1093/jxb/erad347

**Published:** 2023-09-02

**Authors:** Elizabeth Sands, Sian Davies, Richard John Puxty, Valerie Vergé, François-Yves Bouget, David John Scanlan, Isabelle Alice Carré

**Affiliations:** School of Life Sciences, University of Warwick, Coventry, CV4 7AL, UK; School of Life Sciences, University of Warwick, Coventry, CV4 7AL, UK; School of Life Sciences, University of Warwick, Coventry, CV4 7AL, UK; Université Pierre et Marie Curie, Paris 06, UMR 7621, Laboratoire d’Océanographie Microbienne, Observatoire Océanologique, Banyuls sur Mer, France; Université Pierre et Marie Curie, Paris 06, UMR 7621, Laboratoire d’Océanographie Microbienne, Observatoire Océanologique, Banyuls sur Mer, France; School of Life Sciences, University of Warwick, Coventry, CV4 7AL, UK; School of Life Sciences, University of Warwick, Coventry, CV4 7AL, UK; Lawrence Berkeley National Laboratory, USA

**Keywords:** Light quality, niche partitioning, *Ostreococcus*, photophysiology, phytoplankton, picoeukaryote, transcriptomic analysis

## Abstract

Phytoplankton are exposed to dramatic variations in light quality when cells are carried by upwelling or downwelling currents or encounter sediment. We investigated the potential impact of light quality changes in *Ostreococcus*, a key marine photosynthetic picoeukaryote, by analysing changes in its transcriptome, pigment content, and photophysiology after acclimation to monochromatic red, green, or blue light. The clade B species RCC809, isolated from the deep euphotic zone of the tropical Atlantic Ocean, responded to blue light by accelerating cell division at the expense of storage reserves and by increasing the relative level of blue-light-absorbing pigments. It responded to red and green light by increasing its potential for photoprotection. In contrast, the clade A species OTTH0595, which originated from a shallow water environment, showed no difference in photosynthetic properties and minor differences in carotenoid contents between light qualities. This was associated with the loss of candidate light-quality responsive promoter motifs identified in RCC809 genes. These results demonstrate that light quality can have a major influence on the physiology of eukaryotic phytoplankton and suggest that different light quality environments can drive selection for diverse patterns of responsiveness and environmental niche partitioning.

## Teaser

In the photosynthetic picoeukaryote *Ostreococcus*, distinct responses to light quality between two ecotypes are linked to different transcription factor binding sites in the promoters of light-quality responsive genes.

## Introduction

Phytoplankton are a vital part of the marine ecosystem. As primary producers, they provide a source of energy for the food web. They cycle nutrients, form a sink for carbon, and are responsible for roughly half the oxygen released to the atmosphere globally (Kulk *et al.*, 2020). Photosynthetic picoeukaryotes make a major contribution to these processes, being responsible for 25–44% of global carbon capture (Mullin, 2001; Jardillier *et al.*, 2010; Kirkham *et al.*, 2013; Rii *et al.*, 2016). It is therefore important to understand how these organisms adapt to various ecological niches in the ocean, and to identify the environmental factors that influence their abundance. Here, we investigate the effects of light quality, a factor which is generally under recognized in the marine environment.

Whilst the spectrum of incident light must match the absorption spectrum of photopigments for efficient photosynthesis, light quality can also serve as an indicator of competition by other organisms and initiate changes in growth strategy. On land, the light environment is broadly composed of light across the visible spectrum. Land plants possess blue, red, far-red, and UV photoreceptors that enable them to respond to the spectral light environment. While red and blue light both promote photomorphogenesis, a low red to far-red ratio signifies shading by other plants. This triggers the shade avoidance response, which includes elongation of stems and petioles, an increase in leaf area, as well as accelerated flowering (Franklin and Whitelam, 2005). However, distinct factors influence light quality in oceanic systems (Bouman *et al.*, 2000). While visible light in the photosynthetically active radiation (PAR) range reaches a maximum depth of around 200 m, red, far-red, and green light wavelengths are absorbed in the upper layers of the water column, so that only blue light reaches the deep euphotic zone. The light spectrum is also affected by the presence of particulates in coastal waters. Light absorption and scattering by dissolved organic matter and run-off from rivers causes a relative increase in yellow wavelengths (Erga *et al.*, 2012), while abundant phytoplankton increases the proportion of green light (Lichtenthaler, 1987; Tilzer *et al.*, 1995; Schubert *et al.*, 2001; Sun *et al.*, 2010; Kume *et al.*, 2018). Some species of cyanobacteria respond to these changes in light quality through a process known as complementary chromatic adaptation, in which levels of different phycobiliprotein pigments associated with the phycobilisome are adjusted to optimize absorption of excitation energy present in the environment (Sanfilippo *et al.*, 2019). However, much less is known about how eukaryotic microalgae respond to changes in light quality in their environment.

There is mounting evidence that a wide range of species of phytoplankton manifest a response, however. Light quality-dependent changes in photopigments were observed in diatoms, suggesting a form of chromatic adaptation (Brunet *et al.*, 2014; Hintz *et al.*, 2021; Xu *et al.*, 2021). Light quality was also found to affect the protein content of the red alga *Porphyra leucosticte* (Korbee *et al.*, 2005), the diatom *Cyclotella nana*, and the green alga *Dunaliella tertiolecta* (Wallen and Geen, 1971). It influenced the accumulation of lipids in the chlorophyte *Chlorella* sp. and in the heterokonts *Nannochloropsis oculata* and *N. gaditana* (Patelou *et al.*, 2020; Yuan *et al.*, 2020), as well as the growth rates of various phytoplankton species. Most species tested grew the fastest under blue light and the slowest under green light (Patelou *et al.*, 2020; Yuan *et al.*, 2020; Neun *et al.*, 2022), and blue light promoted the highest rate of growth in controlled mesocosm experiments with natural phytoplankton communities (Hintz *et al.*, 2021; Xu *et al.*, 2021). However, *Dunaliella salina* showed the fastest growth rates under red light (Li *et al.*, 2020) suggesting that diverging light quality responses may enable different species or ecotypes of microalgae to thrive in distinct environments, and to occupy different ecological niches.

Here, we investigated the effects of light quality on the transcriptome, pigment content, and photophysiology of *Ostreococcus*, a photosynthetic picoeukaryote found in oceans across the world, including tropical and temperate environments (Rii *et al.*, 2016; Limardo *et al.*, 2017). We show that two ecotypes originating from distinct light quality environments exhibit contrasting responses. These findings suggest that the differential ability to respond to light quality signals may contribute to the specialization of specific phytoplankton ecotypes to different environments.

## Materials and methods

### Cell lines and growth conditions

*Ostreococcus* ecotypes RCC809 (a clone of RCC141) and OTTH0595 (also known as RCC745, RCC4221, OTTH0595, and *O. tauri*) were obtained from the Roscoff Culture Collection (http://roscoff-culture-collection.org). Cells were grown in Keller medium (Keller *et al.*, 1987). Batch cultures were incubated at 21 °C under diurnal light–dark cycles of 12 h light and 12 h darkness from cool-white fluorescent bulbs at an intensity of 20 μmol photons m^−2^ s^−1^. They were transferred to fresh medium every 14 d.

### Monochromatic light sources

Red and blue light was provided by LED arrays, and green light was provided by cool-white, fluorescent bulbs covered with one layer of green filter (Lee filter 139). Light intensity was the same under all conditions, i.e. 4 μmol photons m^−2^ s^−1^. Emission spectra from these different light sources are shown in Supplementary Fig. S1.

### Determination of growth rates

Growth was monitored by measuring absorbance at 550 nm. Cell abundance was estimated based on the equation shown in Supplementary Fig. S2. This relationship was the same for both the OTTH0595 and the RCC809 ecotypes under the different light conditions.

#### Determination of cell size

Flow cytometry measurements of forward scatter signal height were carried out as described previously (Mullaney *et al.*, 1969; Chretiennot-Dinet *et al.*, 1995), 72 h after transfer to monochromatic light. Fluoresbrite Multifluorescent beads with an average diameter of 0.5 μm were used for calibration, in order to allow comparison of relative cell sizes between light conditions.

### RNA-Seq experiment

Cells were grown in 200 ml cultures in 1 litre flasks under white light as above until they reached mid to late log phase (20–40 million cells per ml). They were then transferred to constant monochromatic red, green, or blue light. After 72 h, cells were centrifuged at 5000 *g*, pooled into 1 ml cold phosphate-buffered saline, then spun again at maximum speed in a microfuge. The supernatant was discarded, and the cell pellet frozen in liquid nitrogen. For RNA extraction, 1 ml of TRIzol reagent was added to the frozen samples before thawing at room temperature. Two glass beads were added before shaking for 3 min using a Tissue Lyser (Qiagen) at maximum speed. Chloroform (200 μl) was added and mixed by shaking for 15 s before incubation at room temperature for 3 min and centrifugation at 12 000 *g* at 4 °C for 15 min. The aqueous phase was transferred to a fresh tube, combined with 0.5 ml isopropanol and 5 μl of glycogen (20 mg ml^−1^), incubated for 10 min at room temperature, then spun down at 12 000 *g* at 4 °C for 15 min. The supernatant was removed, and the pellet washed twice with 1 ml 70% (v/v) ethanol before resuspension in 50 μl of RNase-free water. Samples were treated with RNase-free DNase (Sigma-Aldrich) according to the manufacturer’s instructions, before RNA purification using the Spectrum Plant Total RNA Kit (Sigma-Aldrich). RNA quality was verified using a Bioanalyser before preparation of RNA-Seq libraries using the Illumina Tru-Seq RNA library preparation kit, which enriches for mRNAs using Oligo-dT beads to capture polyA tails before cDNA synthesis. This method does not capture chloroplast RNA. One hundred base pair paired-end sequencing was carried out on an Illumina HiSeq at the Oxford Genomics Centre.

RNA-Seq data were analysed within the Cyverse Discovery Environment (https://www.cyverse.org). Contaminating adapter sequences and poor quality sequences were removed using Trimmomatic v 0.36.0 (Bolger *et al.*, 2014). The Fastqc tool version 0.2 was then used to produce fastq files (Andrews, 2015), and TopHat version 2 to map reads to reference genomes using Bowtie 2 (Langmead and Salzberg, 2012; Kim *et al.*, 2013). Samples that gave low numbers of reads were removed from further analyses. CuffDiff version 2.2.1a was used to calculate differential expression values for each gene in each pair of samples (Trapnell *et al.*, 2013). Read counts were normalized by transcript length and by the total number of fragments, using the geometric fragments per kilobase of transcript per million fragments mapped (FPKM) method. Differentially expressed genes were identified based on corrected *P*-values (*Q*-values) and a false discovery rate (FDR) less than 0.05. This gave pairwise comparisons in the form of expression levels sorted by log2-fold change between each pair of light conditions. Gene expression patterns were visualized using the CummeRbund package (Goff *et al.*, 2014), and heatmaps were refined using the Pheatmap package (Kolde, 2019). Reference genomes used were the ORCAE OTTH0595 v2 genome (Derelle *et al.*, 2006; Palenik *et al.*, 2007; Blanc-Mathieu *et al.*, 2014) and the RCC809 v2 genome (Grigoriev *et al.*, 2012). Reference genome sequence (fasta) files and annotation (gff3) files for each *Ostreococcus* ecotype were sourced from the Online Resource for Community Annotation of Eukaryotes (ORCAE) database (http://bioinformatics.psb.ugent.be/orcae/).

### Gene Ontology enrichment analyses and KEGG pathway mapping

OTTH0595 genes were annotated by compiling existing gene descriptions and Gene Ontology (GO) terms from ORCAE and the Universal Protein knowledgebase (UniProt). In order to obtain GO term annotations for RCC809 genes, a genome-wide BLAST search of the NCBI non-redundant database was carried out using the Diamond BLASTx command line tool: ‘translated Query-Protein Subject BLAST 2.2.31+’ (E value 1.00E-03) (Camacho *et al.*, 2009). GO terms were then obtained using BLAST2GO software (Götz *et al.*, 2008). These annotations were compared with and merged with those available from the ORCAE and Interpro databases and with annotations from OTTH0595 homologues, identified by reciprocal BLAST. These were found to be consistent and, in some instances, more detailed than the existing annotations for RCC809. GO term over-representation analyses were carried out using the BiNGO 3.0.3 app in Cytoscape (Maere *et al.*, 2005). Multiple testing correction was carried out using Benjamini–Hochberg FDR correction with a significance cut-off at 0.05.

Pathway mapping using Kyoto Encyclopedia of Genes and Genomes (KEGG) was carried out by searching for the homologues of the RCC809 enzymes in OTTH0595 as RCC809 is not yet listed on KEGG. These homologues were identified using a reciprocal genome-wide BLAST based on a minimum of 50% identity.

### Promoter motif identification

Analysis of the intergenic region length distribution in RCC809 showed a peak around 200 bp. This informed the selection of 250 bp regions upstream of start codons for promoter analyses. Coordinates of these regions were collected in bed files, listing the genomic coordinates for these promoter sequences. Fasta sequence files were then generated from the bed files using the GetFastaBed tool in Galaxy (usegalaxy.org) (Gruening, 2014; Quinlan and Hall, 2010; The Galaxy Community, 2022). Short motifs that were over-represented in the promoters of light-responsive genes compared with a background of promoters genome-wide were identified using the DREME 5.0.4 tool in the MEME suite (http://meme-suite.org) (Bailey, 2011). Matches to known or predicted binding sites for RCC809 transcription factors (TFs) were identified from the CIS-BP database using Motif Scan (Weirauch *et al.*, 2014).

### Analysis of photosynthetic parameters

Cell cultures were grown under white light and then acclimated to monochromatic light for 72 h before analysis of photosynthesis parameters using a PhytoPAM fluorometer. Cultures were diluted 5-fold before analysis to avoid saturation of the fluorescence signal. In order to determine the maximum quantum yield of photosystem II (PSII) photochemistry (*F*_v_/*F*_m_), samples were kept in darkness in the cuvette for 5 min, so that the primary electron acceptor would be fully oxidized and a basal fluorescence level (*F*_0_) could be measured. A saturating light pulse (2600 µmol photons m^−2^ s^−1^ at 470 nm) was then applied at 500 ms intervals and maximum fluorescence (*F*_m_) was measured. To determine the effective quantum yield of PSII photochemistry (Φ_PSII_), samples were subjected to increasing light intensities at 0, 3, 6, 36, 94, 124, 184, 213, 270, and 298 μmol photons m^−2^ s^−1^ at 120 s intervals so that a fluorescence steady state *F*_t_ would be reached. Φ_PSII_ was calculated as (*F*_m_*ʹ*−*F*_t_/*F*_m_*ʹ*) using the Phyto-Win software (V 2.13). The Phyto-Win software also calculated the relative electron transport rate (rETR) as Yield×PAR×0.5 × 0.84 μmol electrons m^−2^ s^−1^, and used the Platt curve fitting model to provide α-values and irradiance at the onset of light saturation (*I*_k_) values from rapid light curve data (Platt *et al.*, 1980). The quantum yield of unregulated non-photochemical energy loss in PSII (Φ_NO_) was calculated as Φ_NO_=*F*/*F*_m_, and the quantum yield of regulated non-photochemical energy loss (Φ_NPQ_) as Φ_NPQ_=(*F*/*F*_m_*ʹ*)−(*F*/*F*_m_) (Klughammer and Schreiber, 2008).

### Pigment analyses

Cell cultures were grown under white light, then acclimated to monochromatic light for 72 h as for the RNA-Seq experiment. Cell cultures were then harvested onto GF/F glass microfibre filters (Whatman) using a vacuum pump. The filters were immediately flash-frozen in cryovials in liquid nitrogen and stored at −80 °C until shipped to the Danish Hydraulic Institute (DHI) laboratory in Denmark for analysis by HPLC as previously described (Van Heukelem and Thomas, 2001). Pigment standards were not available for uriolide, micromonal, and dihydrolutein or for a previously described unknown carotenoid (Guyon *et al.*, 2018), so these were determined using the response factor for β-carotene. The abundance of two chlorophyll *b*-like pigments in RCC809 was calculated using the chlorophyll *b* response factor.

## Results

### Blue light accelerates cell division in the RCC809 ecotype

The RCC809 ecotype of *Ostreococcus* was isolated from the deep euphotic zone of the tropical Atlantic. It is exposed to a range of light conditions in its natural environment, ranging from bright, broad spectrum light near the surface to dim blue light near the bottom of the euphotic zone. While its responses to light intensity have been described previously (Six *et al.*, 2008, 2009), the effects of light quality have not been investigated.

**Fig. 1.**
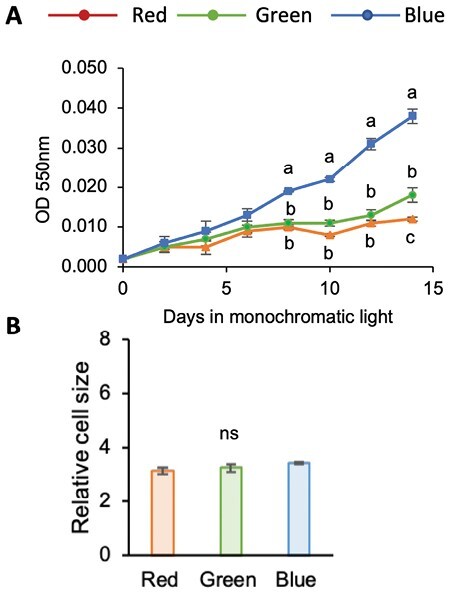
Effects of light quality on RCC809 cultures. (A) Growth kinetics. (B) Cell sizes estimated by forward scatter signal height relative to singlet beads. Cultures were grown under red, blue, or green monochromatic light at 4 μmol m^–2^ s^–1^. Data are means and standard errors from three replicate experiments. Different letters indicate significant differences (*P*<0.05) between light conditions and ns indicates no significant differences, as determined by Student’s *t*-test.

To test the ability of RCC809 to grow under different qualities of monochromatic light, cultures were grown under white light until cell densities reached about 4 × 10^6^ cells ml^−1^, then transferred to red, green, or blue light of equivalent PAR of 4 μmol photons m^−2^ s^−1^. RCC809 cultures grew faster under blue light, reaching cell densities almost twice those under red and green light by the end of the experiment (Fig. 1A).

To test whether the increased biomass under blue light might reflect changes in cell size rather than cell number, flow cytometry measurements of forward scatter signal height (FSC-H) were carried out. FSC-H was previously shown to positively correlate with cell size and was used to assess differences between *Ostreococcus* lineages (Mullaney *et al.*, 1969; Schaum *et al.*, 2016). However, no differences were observed between the different light conditions 72 h after transfer to red, blue, or green light (Fig. 1B), indicating that differences in biomass were solely linked to different rates of cell division.

### Transcriptional responses to light quality in RCC809

**Fig. 2.**
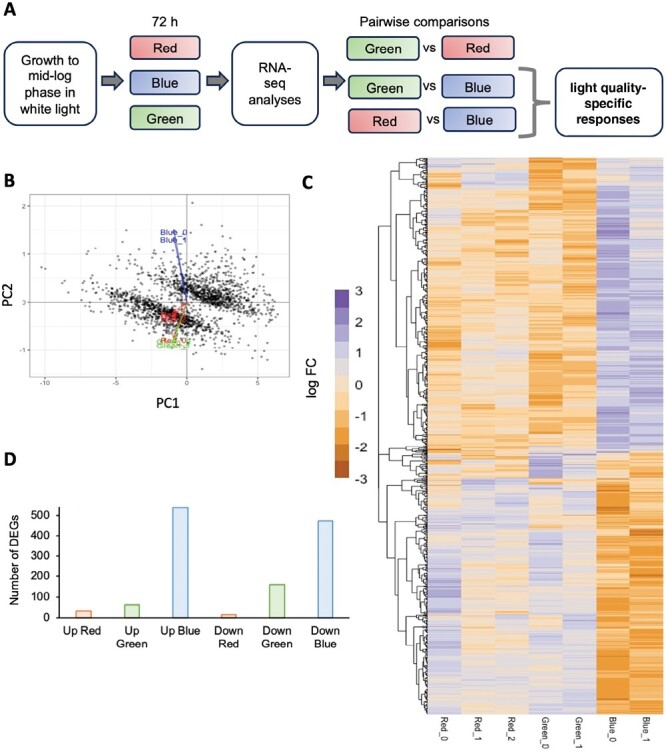
Transcriptomic analysis of RCC809 responses to light quality. (A) Experimental design. Cells were grown to mid-log phase then transferred to red, green, or blue monochromatic light at 4 μmol m^–2^ s^–1^ for 72 h. Cultures were then sampled for RNA-Seq analyses. Light quality-responsive genes were initially identified through pairwise comparison of expression levels between light qualities. Responses specific to individual light qualities were then identified as differences in gene expression that were observed between one light quality and both of the others. (B) Principal component analysis of gene expression data. Red, green, and blue arrows represent the variable vectors for individual samples under the corresponding light conditions. (C) Heatmap of differentially expressed genes (DEGs) identified from the RNA-Seq analysis. Columns correspond to individual samples. Rows correspond to individual genes, clustered by Jensen–Shannon distance. Colour from red to blue indicates log fold changes relative to mean expression levels. (D) Number of genes showing up-regulation (Up) or down-regulation (Down) specific to red, green, or blue light.

To gain insight into which biological processes were affected by light quality, we compared the transcriptomes of cultures that were acclimated to constant monochromatic red, green, or blue light for 72 h (Fig. 2A). Differences in gene expression were analysed by RNA-Seq. Genes that were differentially expressed in pairwise comparisons between light qualities were identified based on corrected *P*-values and FDR less than 0.05 (Table 1; Supplementary Table S1). Principal component analysis (PCA) showed that samples collected under different light qualities clustered into different groups, consistent with differential gene expression (Fig. 2B). Blue light samples were the most distinct, whereas red and green samples were more similar to each other. Thus, the majority of differentially expressed genes had similar expression levels between red and green light conditions, but clearly different expression levels under blue light (Fig. 2C).

**Table 1.** Identification of red, blue, and green light-specific genes by RNA-Seq.

Differential expression	Number of DEGs	Light quality response	Number of light quality-responsive genes
Blue>Red	706	Up–Blue	536
Blue>Green	807	(Blue>Red AND Green)	
Blue<Red	661	Down–Blue	472
Blue<Green	746	(Blue<Red AND Green)	
Red>Blue	666	Up–Red	32
Red>Green	237	(Red>Blue AND Green)	
Red<Blue	706	Down–Red	14
Red<Green	113	(Red<Blue AND Green)	
Green>Blue	746	Up–Green	32
Green>Red	113	(Green>Blue AND Red)	
Green<Blue	807	Down–Green	159
Green<Red	237	(Green<Blue AND Red)	

Differentially expressed genes were first identified in pairwise comparisons between light conditions. Specific light quality responses were then identified as genes that were either up-regulated or down-regulated under one wavelength relative to both others.

To separate gene expression responses by light quality, we identified genes whose expression was significantly increased or decreased under a given wavelength of light, compared with both of the others (Table 1). For example, genes that were induced by blue light were identified as significantly up-regulated in blue light samples relative to both red and green light samples. This identified 536 genes that were specifically induced by blue light, and 472 that were repressed. Only 32 genes were induced and 14 repressed by red light, whereas 60 were induced and 159 repressed by green light (Table 1; Fig. 2D).

Motif over-representation analyses were then carried out to identify candidate regulatory elements responsible for the different types of responses. Potential matches to transcription factor binding sites were identified from the CIS-BP database (Table 2). Remarkably, the most frequent motifs within the promoter sequences of light quality-responsive genes matched binding sites for MYB transcription factors. The sequence GATATTT, found to be over-represented within genes that were down-regulated under red light, matched the known binding site for the MYB transcription factor CCA1 (Od04g00700), a light-responsive component of the *Ostreococcus* circadian clock (Corellou *et al.*, 2009). The motif GGATAG, predicted to be bound by the MYB protein Od06g01160, was over-represented within genes that were induced by red light, as well as within genes that were repressed by blue light. On the other hand, the motif CGATTC, predicted to be recognized by the MYB transcription factor Od12g02060, was over-represented within genes that were up-regulated under blue light. The binding site for the cell cycle-related transcription factor E2F (GTTCCCC) was also over-represented in this group, consistent with the increased rate of cell division observed under blue light. The data further suggested potential roles for the sequence CCACGTGG and for a BHLH transcription factor (Od20g02280) to down-regulate gene expression in response to blue light.

**Table 2.**
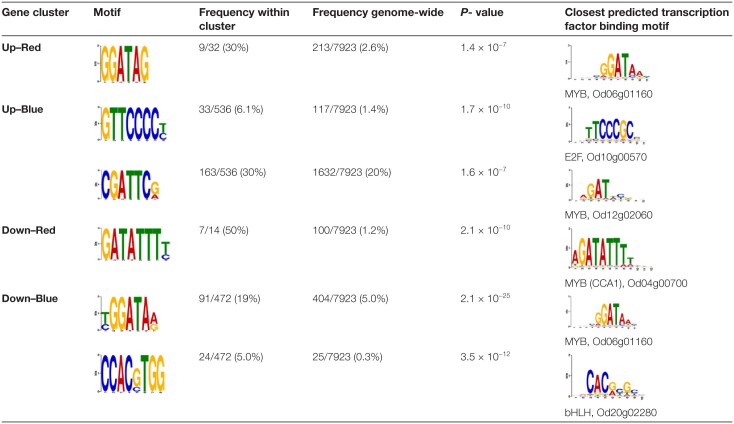
Putative regulatory motifs in the promoters of light quality-responsive genes.

These were identified as over-represented sequences in the 5ʹ upstream region of light quality-responsive genes relative to the whole genome. Cognate transcription factors were identified based on the closest match to a predicted transcription factor binding motif within the CIS-BP database 2.0 (Weirauch *et al.*, 2014).

### Effects of light quality on biological processes in RCC809

GO term analyses were carried out to test for enrichment of specific functional categories amongst light quality-responsive genes, relative to the rest of the genome. Differentially expressed genes were also mapped onto KEGG pathways to reveal potential metabolic consequences. This revealed effects of light quality on pathways related to cell division, primary metabolism, pigment biosynthesis, and photosynthesis (Table 3).

**Table 3.** Overrepresented functional categories in RCC809 light quality-responsive genes.

GO: biological process	Corrected *P*-value	Number of genes (frequency)	Number genome-wide (frequency)
**Down-regulated in green light**			
DNA replication	5.7 × 10^−10^	18 (18%)	89 (2%)
DNA duplex unwinding	1.1 × 10^−2^	5 (4.9%)	24 (0.5%)
Nitrate transport	1.8 × 10^−2^	2 (0.2%)	2 (0.04%)
**Up-regulated in red light**			
NADPH regeneration	2.6 × 10^−3^	4 (16%)	29 (0.7%)
Glycolysis	1.3 × 10^−2^	3 (12%)	33 (0.8%)
Gluconeogenesis	1.45 × 10^−2^	3 (12%)	37 (0.9%)
**Up-regulated in blue light**			
Cellular response to stress	7.3 × 10^−3^	29 (11%)	204 (4.8%)
DNA-dependent DNA replication	7.8 × 10^−3^	11 (4.1%)	42 (1.0%)
Chromosome segregation	1.9 × 10^−2^	7 (2.7%)	21 (0.5%)
Tricarboxylic acid cycle	2.2 × 10^−2^	9 (3.4%)	37 (0.9%)
**Down-regulated in blue light**			
Pigment biosynthetic process	1.6 × 10^−7^	18 (6.6%)	47 (1.1%)
Chl biosynthetic process	9.3 × 10^−7^	11 (5.1%)	19 (0.4%)
Glycolysis	3.9 × 10^−3^	9 (3.3%)	33 (0.8%)
Gluconeogenesis	7.9 × 10^−3^	9 (3.3%)	37 (0.9%)
Carotenoid (tetraterpenoid) biosynthetic process	1.0 × 10^−2^	5 (1.8%)	12 (0.3%)
Nicotinamide nucleotide metabolic process	1.5 × 10^−2^	9 (3.2%)	41 (1.0%)
Lipid biosynthetic process	1.6 × 10^−2^	18 (6.5%)	126 (3.0%)
Photosynthesis	2.4 × 10^−2^	15 (5.5%)	101 (2.4%)
Glucan biosynthetic process	4.7 × 10^−2^	4 (1.5%)	11 (0.3%)
Reductive pentose-phosphate cycle	4.7 × 10^−2^	2 (0.7%)	2 (0.05%)
Endosome organization	4.7 × 10^−2^	2 (0.7%)	2 (0.05%)
Phylloquinone biosynthetic process	4.7 × 10^−2^	2 (0.7%)	2 (0.05%)

Forty-one genes related to the cell division cycle were differentially expressed under the different light qualities, including genes related to DNA replication, DNA repair, and chromosome segregation (Supplementary Table S1). Of these, 27 were up-regulated under blue light relative to red and green light (Supplementary Table S1). For example, the expression of cyclin-dependent kinase B (Od14g01080), which is up-regulated during S-phase in *Ostreococcus* (Corellou *et al.*, 2005; Farinas *et al.*, 2006), more than doubled under blue light (Supplementary Fig. S3), and expression of two homologues (Od08g01730 and Od06g06460) of the Rad51 recombinase, which plays a role in double strand repair during DNA replication (Lim *et al.*, 2020), was up-regulated in a similar manner. These observations were consistent with the faster growth rates observed in blue light relative to red and green light and suggested that exposure to blue light signals the acceleration of the cell cycle in the RCC809 ecotype of *Ostreococcus*.

The RNA-Seq analysis also revealed changes in expression of genes with key roles in photosynthetic carbon fixation. Three different genes encoding the small subunit of RuBisCO (Od17g01990, Od17g02000, and Od17g02010) showed a 2-fold reduction in expression under blue light relative to red light, with intermediate levels being observed under green light (Fig. S4A-C). Rubisco activase (Od04g02820), which enhances Rubisco activity through removal of competitive inhibitors (Portis, 2003), showed similar trends (Supplementary Fig. S4D) suggesting that exposure to blue light may lead to less efficient carbon fixation.

In addition, multiple enzymes in the glycolysis and gluconeogenesis pathways were down-regulated under blue light (Supplementary Fig. S5). While most of these enzymes had both catabolic and anabolic functions, the down-regulation of four different fructose-1,6-bisphosphatase genes (Od09g06250, Od06g06980, Od03g02850, and Od20g01100) under blue light suggested a decrease in gluconeogenesis in this condition, while up-regulation of pyruvate dehydrogenase suggested an increased rate of glycolysis. Genes associated with fatty acid biosynthesis were also down-regulated under blue light (Supplementary Fig. S6), whereas multiple enzymes in the tricarboxylic acid (TCA) cycle were up-regulated under blue light. This included citrate synthase (Od05g01850), which catalyses the first step of the cycle, and three genes encoding malate dehydrogenase (Od03g03160, Od06g02230, and Od08g00070), which regenerates oxaloacetate from malate (Supplementary Fig. S7). Altogether, these results suggested an increased rate of catabolism and a reduction in energy storage under blue light.

The gene expression data further suggested that synthesis of photopigments (chlorophylls and carotenoids) was globally reduced under blue light, relative to red and green light. Seven genes encoding enzymes in the plastidic 2-*C*-methyl-d-erythritol 4-phosphate/1-deoxy-d-xylulose 5-phosphate (MEP/DOXP) terpenoid backbone biosynthesis pathway showed reduced expression levels under blue light, while only one showed lower expression under green light (Supplementary Fig. S8). Multiple enzymes with roles in synthesis of porphyrin precursors of chlorophylls were also down-regulated under blue light (Supplementary Fig. S9A), including the enzyme glutamate-1-semialdehyde aminotransferase (GSA-AM) HemL (Od19g01200), which catalyses the production of 5-amino levulinic acid (dALA) from glutamate-1-semialdehyde and is rate-limiting in Arabidopsis (Sinha *et al.*, 2022).

Expression of chlorophyll *a* (Chl *a*) synthase (Od08g01040) was down-regulated under blue light (Supplementary Fig. S9B). As this enzyme catalyses the last step of Chl *a* biosynthesis, this suggested potential shifts in the ratio of Chl *b* to Chl *a*. Furthermore, the expression of violaxanthin de-epoxidase (Od11g00170), which catalyses the conversion of violaxanthin to zeaxanthin, was elevated under blue light, whereas zeaxanthin epoxidase (Od02g02570), which catalyses the reverse reaction, was elevated under red light (Supplementary Fig. S10A, B). A member of the CYP97 carotene hydroxylase family (Od09g03950) showed reduced expression under blue light compared with red light (Supplementary Fig. S10C). As this enzyme plays a role in the conversion of carotenes to lutein (Cui *et al.*, 2013), this suggested that the synthesis of lutein and its derivatives may decrease under blue light.

Changes related to photosystems were also observed. Two genes with roles in the synthesis of phylloquinone (Od07g04840 and Od20g02670) were down-regulated under blue light. Phylloquinone acts as a cofactor in photosystem I (PSI)-mediated electron transport between the primary electron acceptor and ferredoxin, and a decrease in phylloquinone was previously shown to be associated with a decrease in PSI activity (Gross *et al.*, 2006). Six genes predicted to encode PSII assembly proteins showed also elevated expression under blue light, suggesting an increase in PSII content, but expression of another PSII component was reduced, suggesting a possible change in composition (Supplementary Table S2).

*Ostreococcus* possesses multiple photoreceptors, including a phototropin, a light–oxygen–voltage histidine kinase (LOV-HK), three cryptochromes, and a histidine kinase rhodopsin (HKR) (Heijde *et al.*, 2010; Djouani-Tahri *et al.*, 2011; Sullivan *et al.*, 2016; Luck *et al.*, 2019). While genes involved in light perception were not found to be significantly over-represented within any group of light quality-responsive genes, both genes encoding LOV-HK (Od09g04310 and Od09g04300) showed reduced expression under blue light compared with red light (Supplementary Fig. S11). The phototropin photoreceptor (Od15g00300) and the cryptochrome OdCPF1 (Od05g00310) showed a similar response. In contrast, expression of the Cry-DASH cryptochrome OdCPF2 (Od01g00210) was elevated in blue light, whereas expression of the histidine kinase rhodopsin (HKR) photoreceptor (Od15g00300) was not affected by light quality.

### Changes in pigment content and photosynthetic parameters in RCC809

**Fig. 3.**
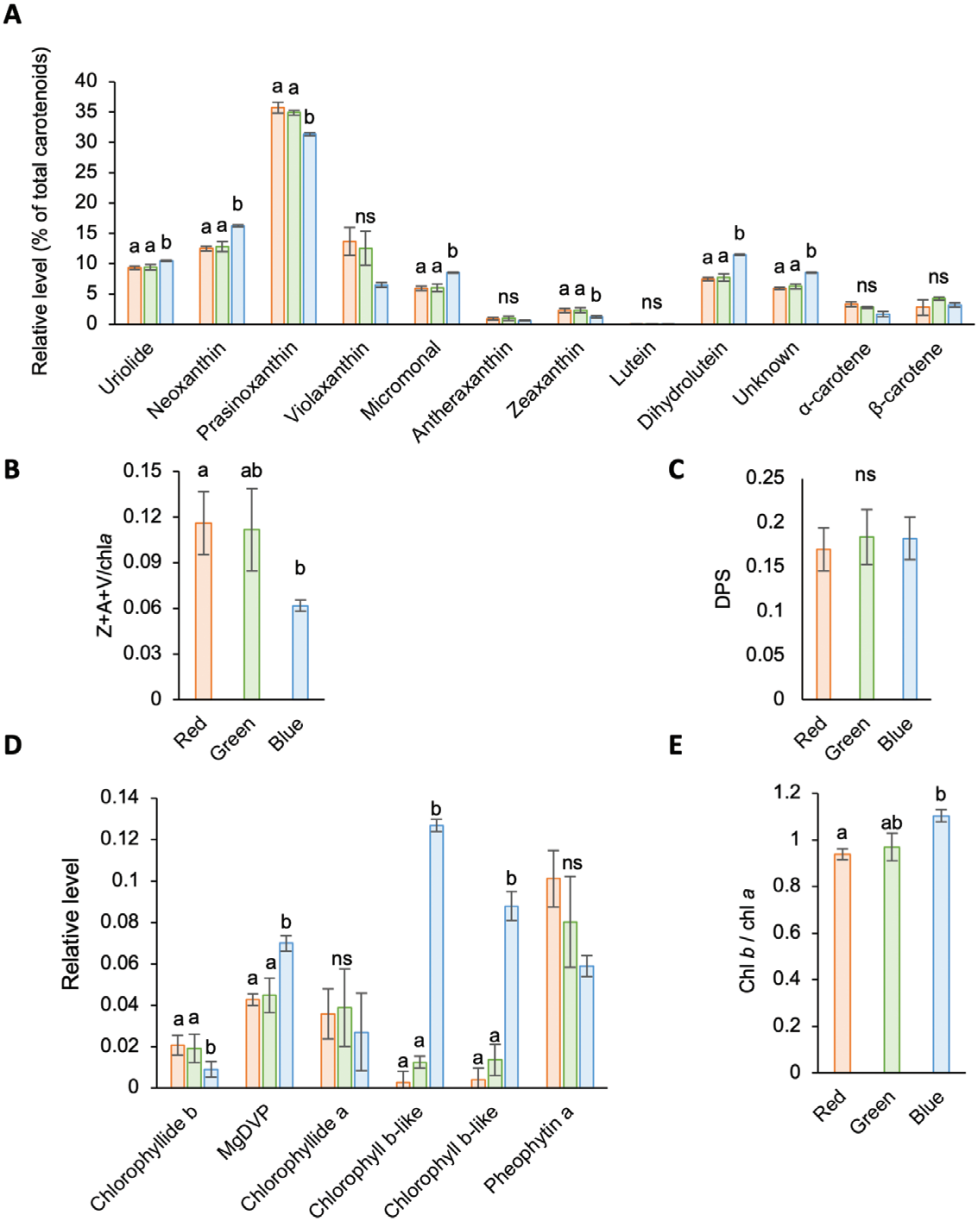
Analysis of pigment contents in RCC809 following acclimation to different light qualities. (A) Carotenoid pigments. (B) Total xanthophylls relative to chlorophyll *a* (Chl *a*). Z, A, and V correspond to zeaxanthin, antheraxanthin, and violaxanthin, respectively. (C) Xanthophyll depoxidation state (DPS), calculated as (Z+0.5A)/(V+A+Z). (D) Chlorophylls and chlorophyll derivatives. Levels are expressed relative to Chl *a*. (E) Chl *a* to Chl *b* ratio. Red, green, and blue bars correspond to samples acclimated to red, green, and blue monochromatic light for 72 h. Data are means and standard errors from triplicate experiments. Different letters indicate significant differences (*P*<0.05) between light conditions and ns indicates no significant differences, as determined by Student’s *t*-test.

To uncover the functional consequences of these changes in gene expression, HPLC analyses of cell pigment contents were carried out. This revealed elevated levels of several carotenoids under blue light relative to red and green light, including neoxanthin, micromonal, dihydrolutein, uriolide and a previously described unknown carotenoid (Guyon *et al.*, 2018). In contrast, prasinoxanthin, violaxanthin, and zeaxanthin levels were reduced (Fig. 3A).

Carotenoids and their oxygenated xanthophyll derivatives play important roles within light-harvesting complexes, both as accessory light-harvesting pigments and to protect the photosynthetic apparatus from damage by excessive light. The conversion of violaxanthin (V) to antheraxanthin (A) and zeaxanthin (Z) through the de-epoxidation reactions of the xanthophyll cycle acts to protect PSII from light damage through quenching of excessive excitation energy and dissipation into heat, a process known as non-photochemical quenching (NPQ) (Goss and Latowski, 2020). The xanthophyll cycle can also play a role in detoxification of reactive oxygen species (ROS) which are generated under supersaturating light conditions. To assess the potential for xanthophyll cycle induction for photoprotection, the total xanthophyll to Chl *a* ratio was calculated as Z+A+V/Chl *a*, where Z, A, and V correspond to relative levels of zeaxanthin, antheraxanthin, and violaxanthin, respectively (Demmig-Adams, 1990). This ratio was smaller under blue light than in red or green light, indicating a reduction in the total xanthophyll pool size under blue light relative to red or green light. (Fig. 3B). However, no differences were noted in the depoxidation state, calculated as (Z+0.5A)/(V+A+Z) (Munné-Bosch and Cela, 2006) (Fig. 3C).

As predicted by the gene expression data, blue light induced an increase in Chl *b* levels relative to Chl *a* (Fig. 3D, E). However, the most remarkable response to blue light was an approximately 10-fold increase in two unknown pigments, which were annotated as Chl *b*-like based on their spectral similarity to Chl *b* (Fig. 3D; Supplementary Fig. S12). The chlorophyll metabolite Mg-2,4-divinyl pheoporphyrin (MgDVP), which plays a role as a light harvesting accessory pigment, also increased significantly relative to Chl *a*, almost doubling in blue light compared with both red and green light (Fig. 3D). Altogether these results demonstrated that light quality influences the pigment composition of the RCC809 strain of *Ostreococcus*, with potential consequences for light-harvesting efficiency.

**Fig. 4.**
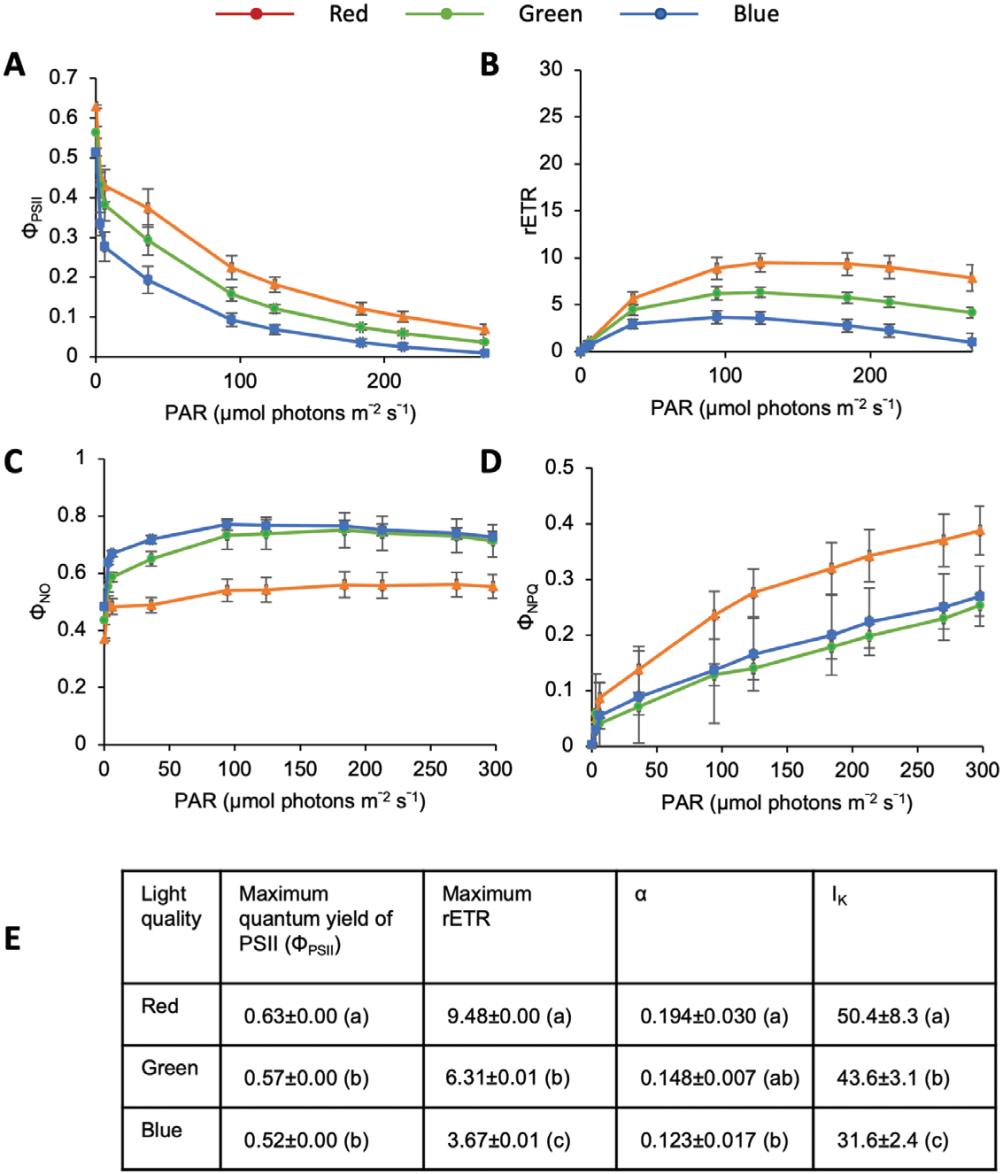
Effect of light quality on photosynthetic parameters in RCC809. (A–D) Rapid light curves showing (A) the quantum yield of Photosystem II (Φ_PSII_), (B) the relative electron transport rate (rETR), (C) the quantum yield of unregulated non-photochemical energy loss in PSII (Φ_NO_), and (D) the quantum yield of regulated non-photochemical energy loss in PSII (Φ_NPQ_) as a function of irradiance (photosynthetically active radiation (PAR)) for cultures acclimated to red, green, or blue light. Φ_PSII_+Φ_NPQ_+Φ_NO_=1. (E) Derived photosynthetic parameters, including the maximum quantum yield, maximum rETR, initial slope of the rETR curve (α), and irradiance at the onset of light saturation (*I*_k_). Data are the mean and standard error from triplicate experiments. Different letters indicate significantly different values (*P*<0.05) as determined using Student’s *t*-test.

To test the consequences of these pigment responses for photosynthetic activity, pulse-amplitude modulation fluorometry analysis was used to compare the efficiency of PSII after acclimation to the different light qualities. We first assayed the quantum yield of PSII photochemistry (Φ_PSII_) under short pulses of increasing light intensity at 470 nm (Ralph and Gademann, 2005). As expected, the quantum yield of PSII was highest in response to low intensity light pulses where carbon fixation operates at maximum photosynthetic efficiency, and decreased at higher light intensities as electron transfer pathways became saturated (Fig. 4A). While similar changes were observed in cells adapted to different light conditions, Φ_PSII_ was always highest in cells acclimated to red light and lowest in cells acclimated to blue light at a given intensity of excitatory light.

The relative electron transport rate (rETR) increased with the intensity of excitation, reaching a plateau around 50–150 μmol photons m^−2^ s^−1^, then decreased at higher light intensities (Fig. 4B). Higher electron transfer rates were observed in cells acclimated to red light, and lower rates in cells acclimated to blue light. Furthermore, saturation was reached at a lower intensity in cells acclimated to blue light than in cells acclimated to red light (about 30 and 50 μmol photons m^−2^ s^−1^, respectively, as indicated by *I*_k_ values in Fig. 4E). These findings suggest that the photosynthetic machinery in RCC809 can utilize light energy more efficiently after acclimation to red light as compared with blue light. Cells acclimated to green light showed an intermediate phenotype.

Photoprotection processes were also impacted by light quality. Consistent with the increased total xanthophyll pool size under red light, Φ_NPQ_ values were elevated under this light condition (Fig. 4C), indicating an increased capacity for photoprotection through processes regulated by the transthylakoid proton gradient (Kramer *et al.*, 2004). Conversely, Φ_NO_ values, which quantify energy dissipation through unregulated processes, were highest under blue light (Fig. 4D). This suggested that cells acclimated to this wavelength were less able to protect themselves against damage by excess illumination.

Φ_PSII_ and rETR values under green light were intermediate between those under red and blue light (Fig. 4A, B). However, Φ_NPQ_ and Φ_NO_ values under green light were similar to those under blue light (Fig. 4C, D), suggesting that green light did not induce photoprotection in spite of the increased xanthophyll pool size relative to blue light.

### Light quality responses are attenuated in OTTH0595, a shallow water ecotype of *Ostreococcus*

While light intensity is thought to be the primary abiotic factor determining the clade distribution of *Ostreococcus* species (Rodriguez *et al.*, 2005; Six *et al.*, 2009; Demir-Hilton *et al.*, 2011; Botebol *et al.*, 2017), we hypothesized that light quality may also play a role. Our prediction was that an ecotype originating from a shallow water environment and exposed to the full visible light spectrum at all times may exhibit distinct responses to light quality from a deep ocean ecotype, which is exposed to variable wavelengths of light as it is moved up and down the water column. We therefore compared the light quality responses of the deep ocean ecotype RCC809 with those of the lagoon species OTTH0595, also known as *O. tauri*.

**Fig. 5.**
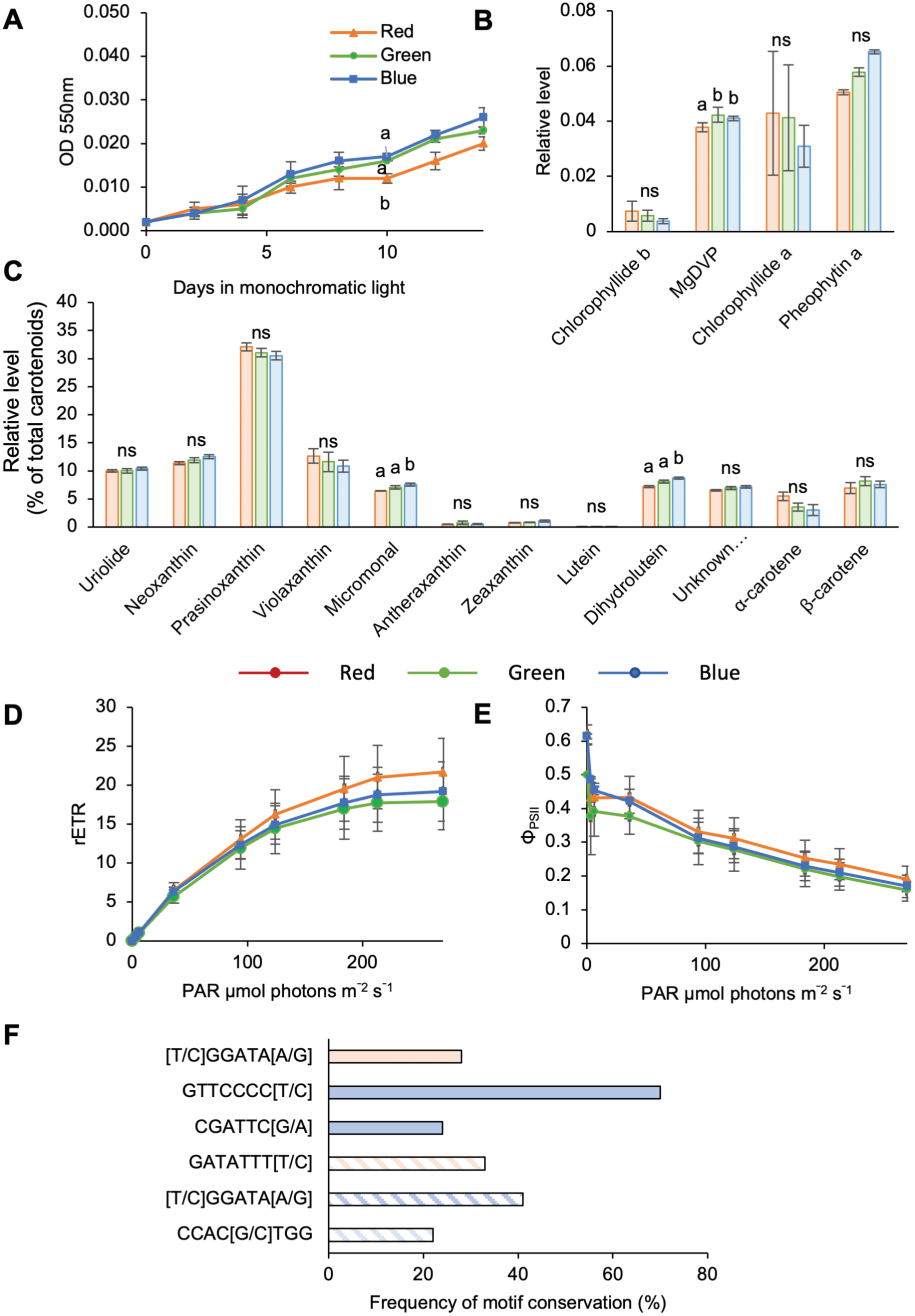
Light quality responses in the lagoon species OTTH0595, also known as *O*. *tauri*. (A) Growth kinetics. (B, C) Levels of chlorophylls and chlorophyll derivatives relative to Chl *a*, and levels of carotenoid pigments relative to total carotenoids. Red, green, and blue bars correspond to samples acclimated to red, green, and blue monochromatic light for 72 h. Data are the mean and standard error from triplicate experiments. Different letters indicate significant differences (*P*<0.05) between light conditions and ns indicates no significant differences, as determined by Student’s *t*-test. (D, E) Photosynthetic light-response curves showing the quantum yield of photosystem II (Φ_PSII_) and relative electron transport rate (rETR) as a function of irradiance (photosynthetically active radiation (PAR)), for cultures acclimated to red, green, or blue light. Data are the mean and standard error from triplicate experiments. (F) Frequency of motif conservation between RCC809 red and blue light-responsive genes and their OTTH0595 homologues. Solid red and blue bars correspond to genes up-regulated under red and blue light, respectively. Hatched red and blue bars correspond to genes down-regulated under red and blue light.

OTTH0595 growth rates were similar under all light qualities and only slightly lower under red light compared with blue and green light (Fig. 5A). HPLC analyses revealed that the Chl *b*: Chl *a* ratio was not affected by light quality (Fig. 5B). Small increases in micromonal and dihydrolutein contents were observed in blue light (9% and 14%, respectively; Fig. 5C), but these were minor changes compared with those observed in RCC809 (43% and 52%, respectively). Consistent with previous observations that OTTH0595 tolerates higher light intensities (Cardol *et al.*, 2008; Six *et al.*, 2008, 2009; Demir-Hilton *et al.*, 2011), rETR values were higher than in RCC809 and did not decrease at high light intensities. However, no significant differences in PSII quantum yield or rETR were observed between light qualities (Fig. 5D, E).

To examine whether the attenuated light responses of the OTTH0595 species were linked to divergence in gene regulation relative to RCC809, we identified the OTTH0595 homologues of light quality-responsive genes from RCC809 and examined their upstream regulatory sequences for the presence or absence of the candidate regulatory elements identified in Table 2. While the GTTCCCC[T/C] motif, recognized by the cell cycle-related transcription factor E2F, was conserved in 80% of OTTH0595 homologues, other motifs were only present in 30–40% of OTTH0595 genes, suggesting that they either had been lost in OTTH0595 or gained in RCC809 during the course of their evolutionary divergence (Fig. 5F).

## Discussion

### Blue light signals a distinct growth strategy from red and green light in RCC809

Our results demonstrate that the *Ostreococcus* ecotype RCC809 exhibits responses to light quality. Transcriptomic analyses revealed differences in gene expression between cells acclimated to red, green, and blue monochromatic light (Fig. 2B, C). Gene expression under blue light was most distinct from that under red light, whilst intermediate phenotypes were seen under green light. *Ostreococcus* responses to light quality contrasted with those of land plants, where the effects of green light oppose those of red and blue wavelengths (Wang and Folta, 2013). This distinct pattern of light quality responses may be linked to the presence of a different set of photoreceptors. While cryptochrome and phototropin photoreceptors are present across all plants and algae (Kianianmomeni and Hallmann, 2014), *Ostreococcus* contains the histidine kinases LOV-HK and HKR, which are absent in land plants, but does not contain phytochomes. LOV-HK and HKR may be responsible for responses to blue and green light, respectively (Djouani-Tahri *et al.*, 2011; Luck *et al.*, 2019), but the identity of the photoreceptor responsible for perception of red light remains unclear.

**Fig. 6.**
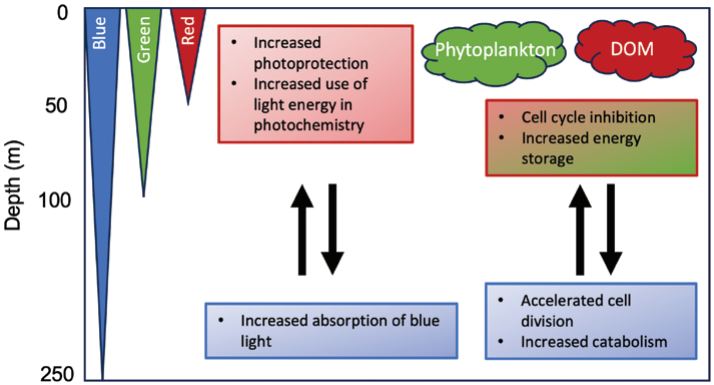
Conceptual model showing how the RCC809 ecotype of *Ostreococcus* may alter its physiology in response to changes in light quality as it is moved up and down the water column. The colour of text boxes indicates the specific wavelengths inducing the responses. Coloured wedges on the left represent the decrease in red, green, and blue light wavelengths with increasing depth. Perception of red may signal proximity to the surface. This may enable cells to anticipate exposure to high light intensities by increasing their potential for photoprotection as well as their ability to utilize light energy. Further, increased production of blue light-absorbing pigments may maximize light capture in the lower part of the euphotic zone where only blue wavelengths can penetrate. Green and red clouds on the right represent increased proportions of green and red wavelengths, caused by the presence of phytoplankton, dissolved organic matter (DOM) and other sediments in shallow coastal waters. Detection of these wavelengths may be interpreted as unfavourable conditions for growth and lead to slower cell division.

Blue light signalled a switch in physiology and metabolism to a state that favoured growth at the expense of energy storage (Fig. 6). Thus, acclimation to blue light resulted in the elevated expression of genes associated with the cell cycle and faster growth rates (Fig. 1; Supplementary Fig. S3). Conversely, expression of enzymes involved in starch or fatty acid biosynthesis was reduced in blue light, whereas the expression of TCA cycle enzymes was elevated (Supplementary Figs S5–S7). Exposure to green or red light elicited the opposite response.

We questioned whether the gene expression signatures observed under green light might represent a starvation response due to poor absorption of light by chlorophylls in this part of the spectrum. However, the fact that similar responses were observed under red and green light argued against this, because red light is absorbed efficiently by chlorophylls (Liu and van Iersel, 2021). Furthermore, the growth arrest observed under red or green light (Fig. 1) was accompanied by up-regulation of starch and fatty acid biosynthesis pathways (Supplementary Figs S5–S7), which is not consistent with a starvation response. We suggest that enrichment of red or green wavelengths may signal conditions less favourable for growth in the natural environment, such as shading by sediments or other particulate material, competition from other organisms, or exposure to damaging light intensities near the surface.

### RCC809 adjusts its pigment contents and photosystem function as a function of the light quality environment

Previous work showed that increases in light intensity induce the down-regulation of both Chl *a* and Chl *b* content, accumulation of lutein, and increased xanthophyll de-epoxidation in the RCC809 ecotype of *Ostreococcus* (Six *et al.*, 2008). Blue light triggered a distinct set of responses in our experiment, suggesting that cells did not respond to the higher energy levels carried by blue wavelengths, but rather to the specific light quality.

Blue light induced an increase in the carotenoid pigments dihydrolutein and micromonal, as well as an increase in MgDVP, Chl *b* and Chl *b*-like pigments relative to Chl *a* (Fig. 3A, D). This was consistent with chromatic adaptation, as Chl *b* and MgDVP absorb much more under blue light than Chl *a* and these changes would be expected to enhance cells’ ability to utilize blue light for photosynthesis (Raven, 1996).

In contrast, red and green light induced expression of enzymes involved in the biosynthesis of Chl *a* (Supplementary Fig. S9), which would be predicted to increase light absorption under red light. Whilst the lower Chl *b*: Chl *a* ratio suggested that the size of the photosynthetic antenna was smaller in cells acclimated to red light (Fig. 3E), PSII efficiency was increased as indicated by higher PSII quantum yield and rETR values (Fig 4A, B). This may be linked to higher Rubisco activity, as the expression of Rubisco small subunits and of Rubisco activase was higher under red and green light, compared with blue light (Supplementary Fig. S4), and the Rubisco activation state is known to be positively correlated with the electron transport rate in higher plants (Perdomo *et al.*, 2017).

An increase in the total xanthophyll pool size was also observed under red and green light (Fig. 3B), indicating an increased potential for photoprotection. Consistent with this observation, red or green light-adapted cultures maintained higher PSII quantum yields and rETR under increasing light intensities (Fig. 5A, B). However, increased Φ_NPQ_ values were only observed following acclimation to red light, indicating that only red light induced photoprotection (Fig. 5D). This suggests that while both red and green light signal unfavourable growth conditions, perception of red light may specifically signal proximity to the surface and allow cells to anticipate exposure to high light intensities through induction of photoprotective mechanisms (Fig. 6).

### Evidence for adaptation to different light quality environments

We compared the light quality responses of the RCC809 ecotype, isolated from the tropical Atlantic Ocean at a depth of 105 m, with those of the lagoon ecotype OTTH0595. These two *Ostreococcus* species were previously shown to belong to distinct clades (Demir-Hilton *et al.*, 2011) and to exhibit distinct responses to light intensity (Cardol *et al.*, 2008; Six *et al.*, 2008, 2009). OTTH0595 originates from shallow environments where it is not greatly affected by the narrowing of spectral availability associated with depth (Potes *et al.*, 2013). Instead, the spectral quality varies mostly according to the level of suspended sediment and other photosynthetic organisms. It may therefore be less important for this species to be able to perceive the blue-light-enriched environment associated with depth, and to adjust its photosystems accordingly. Furthermore, OTTH0595 showed no differences in pigment content when adapted to different light qualities, except for a small increase in dihydrolutein and micromonal under blue light, and its PSII quantum yield and rETR were unaffected (Fig. 5A–E).

While OTTH0595 possesses the same set of photoreceptors as RCC809, mechanistic clues to their distinct responses to light quality may be obtained through investigation of promoter motifs and their cognate transcription factors. Analysis of over-represented motifs within RCC809 light quality-responsive promoters identified a number of candidate regulatory motifs, including binding sites for MYB and bHLH transcription factors. Remarkably, most of these promoter motifs were missing from OTTH0595 homologues of RCC809 light quality-responsive genes (Fig. 5F), suggesting a lack of selective pressure for such regulation in OTTH0595. Furthermore, a single bHLH transcription factor was identified in OTTH0595, as compared with four in RCC809, suggesting the loss of light-responsive transcription factors. Our findings suggest that distinct light quality environments in the aquatic environments can result in different evolutionary pressures and may select for gain or loss of regulatory connections downstream of photoreceptors.

### Perspectives

Taken together, these observations suggest that, whilst light intensity has been considered the primary abiotic factor determining the distribution of *Ostreococcus* ecotypes, differences in light quality responses of ecotypes should also be considered as an important factor in determining adaptation to distinct environmental niches. Further work will be required, however, to determine whether our observations can be generalized to other *Ostreococcus* species. These were previously classified into four different clades, based on small ribosomal RNA gene sequences (Guillou *et al.*, 2004; Subirana *et al.*, 2013). RCC809 and OTTH0595 (also known as *O. tauri*) belong to clades B and C. Clades A and D include *O. lucimarinus* and *O. mediterraneus*, respectively (Worden *et al.*, 2004; Subirana *et al.*, 2013), and are associated with relatively shallow coastal zones (Demir-Hilton *et al.*, 2011; Limardo *et al.*, 2017). While the distribution of these species was previously proposed to be driven by differences in temperature, salinity, nutrient abundance, and light intensity, our findings suggest that light quality may also contribute. Based on the similarity of their light environment to OTTH95, clade B and C species are predicted to exhibit very limited responses to light quality.

It remains unclear how *Ostreococcus*’ responses to light quality contribute to fitness in different environments. Future metatranscriptomic analyses may provide valuable clues by revealing where these responses are observed in nature. We hypothesize that what was described here as responses to red or green light may be observed near the surface, whereas blue light responses would be observed at depth. However, such analyses are likely to be complicated by varying light intensities, and the fact that cells in the natural environment are exposed to the full spectrum of light.

It also remains to be determined whether our findings in *Ostreococcus* RCC809 apply to other types of eukaryotic phytoplankton. While few detailed transcriptomic analyses are available, there is mounting evidence that other species of microalgae exhibit responses to light quality, affecting carbon metabolism and lipid accumulation (Lehmuskero *et al.*, 2018; Maltsev *et al.*, 2021), pigment composition (Rochet *et al.*, 1986; Valle *et al.*, 2014; Wei *et al.*, 2020; Maltsev *et al.*, 2021), and rates of cell division (Neun *et al.*, 2022). The specific responses vary between organisms, however. For example, the diatom *Phaeodactylum tricornutum* (CCMP2561) grew faster under blue light than under red light (Huysman *et al.*, 2013). This was associated with induction of the cell cycle gene *diatom-specific cyclin2* (*DSCYC2*) through activation of the diatom-specific photoreceptor aureochrome and of the cryptochrome/photolyase CPF1 (Coesel *et al.*, 2009; Huysman *et al.*, 2013). Blue light also promoted the growth of the green algae *Chlorella* sp. HQ (Liu *et al.*, 2021) and *Chlorella pyrenoidosa* (Guo and Fang, 2020). In contrast, yellow and red wavelengths were reported to promote faster growth of *Chlorella vulgaris* and *Dunaliella salina* than blue light (Hultberg *et al.*, 2014; Li *et al.*, 2020),

Light quality was found to affect photoprotective responses in a broad range of microalgae. Blue light induced accumulation of chlorophyll and carotenoid pigments in *Chlorella* sp. HQ (Liu *et al.*, 2021), *Chlamydomonas reinhardtii* (Redekop *et al.*, 2022), *Nannochloropsis oceanica* (Wei *et al.*, 2020), *Dunalliella salina* (Li *et al.*, 2020), and *Phaeodactylum tricornutum* strain IO 108-01 (Duarte *et al.*, 2021). Blue light induced expression of the photoprotective genes *LHCSR1*, *LHCSR3.1*, and *PSBS1* in *Chlamydomonas reinhardtii* (Redekop *et al.*, 2022), and the induction of energy-dependent quenching (qE quenching) required phototropin, a blue light photoreceptor (Petroutsos *et al.*, 2016; Aihara *et al.*, 2019; Redekop *et al.*, 2022). Blue light also promoted expression of photoprotective, ROS-scavenging enzymes in *Dunaliella salina* (Li *et al.*, 2020). In contrast, ROS scavenging pathways were up-regulated under red light in *Nannochloropsis oceanica* (Wei *et al.*, 2020). In *Phaeodactylum tricornutum*, different studies showed induction of photo protective responses by different light qualities, and these apparent discrepancies may in fact reflect differences between strains. Thus, blue light induced an increase in xanthophyll pool size in strain UTEX 646 (Schellenberger Costa *et al.*, 2012) and the expression of genes involved in repair of photodamaged PSI and ROS scavenging in strain CCMP632 (Valle *et al.*, 2014). However, red light induced accumulation of fucoxanthin in strain IO 108-01, resulting in a higher potential for photoprotection than under blue light (Duarte *et al.*, 2021). These observations, together with the results presented here, suggest that differences in light quality responses between eukaryotic microalgae can arise within relatively close taxonomic groups.

Our findings suggest that light quality in the aquatic environment represents a significant evolutionary pressure that can lead to extensive rewiring of transcriptional responses downstream of photoreceptors. The suppression of some responses and the acquisition of others through loss or gain of transcription factor binding sites in the promoters of light-responsive genes may allow different phytoplankton species to differentiate their light quality responses from each other, enabling the colonization of distinct niches in the environment. The spectral tuning of photoreceptors and their loss in some algal lineages may also contribute to adaptation to different aquatic environments (Jaubert *et al.*, 2017). Changes in ocean colour resulting from climate change will lead to ecosystem changes (Cael *et al.*, 2023) and it will be important to understand how light quality responses contribute to the fitness of different phytoplankton species, in order to predict the consequences of these changes for the global distribution of these primary producers in the oceans and for the aquatic food chain.

## Supplementary data

The following supplementary data are available at *JXB* online.

Fig. S1. Spectroradiometer analysis of the three light conditions used in our experiments.

Fig. S2. Validation of the use of absorbance at 550 nm (OD 550 nm) to estimate the cell abundance of *Ostreococcus* cultures.

Fig. S3. Examples of cell cycle-related genes up-regulated under blue light.

Fig. S4. Effect of light quality on the expression of Calvin cycle-related genes.

Fig. S5. Effect of light quality on the expression of glycolysis and starch synthesis-related genes.

Fig. S6. Effect of light quality on the expression of fatty acid synthesis-related genes.

Fig. S7. Effect of light quality on the expression of TCA cycle-related genes.

Fig. S8. Effect of light quality on the expression of terpenoid biosynthesis-related genes.

Fig. S9. Effect of light quality on the expression of porphyrin and chlorophyll metabolism-related genes.

Fig. S10. Effect of light quality on the expression of carotenoid biosynthesis-related genes.

Fig. S11. Effect of light quality on the expression of *Ostreococcus* RCC809 genes encoding photoreceptors.

Fig. S12. Examples of HPLC chromatograms illustrating the range of pigments detected in the (A) RCC809 and (B) OTTH0595 ecotypes of *Ostreococcus*.

Table S1. Lists of differentially expressed genes identified for RCC809 in pairwise comparisons between red and green, red and blue, or blue and green light conditions.

Table S2. Light quality-responsive genes related to the cell cycle.

Table S3. Light quality-responsive genes with roles in photosystem II assembly.

erad347_suppl_Supplementary_Figures_S1-S12_Tables_S1-S3

## Data Availability

The transcriptomics dataset was deposited in the Gene Expression Omnibus database under accession number GSE221420.
