# Supplementary material for: Genetic and physiological responses to light quality in a deep ocean ecotype of Ostreococcus, an ecologically important photosynthetic picoeukaryote
Source: J Exp Bot. 2023 Sep 2;74(21):6773–89. doi: 10.1093/jxb/erad347 (PMC10662239; doi:10.1093/jxb/erad347)
Supplement: erad347_suppl_Supplementary_Figures_S1-S12_Tables_S1-S3 [file erad347_suppl_supplementary_figures_s1-s12_tables_s1-s3.pdf]

Supporting Information for: ***Genetic and physiological responses to light quality in* a deep ocean ecotype of *Ostreococcus, an ecologically important photosynthetic picoeukaryote***

Authors: Elizabeth Sands, Sian Davies, Richard J. Puxty, Valerie Vergé, François-Yves Bouget, David J. Scanlan and Isabelle Alice Carré

The following Supporting Information is available for this article:

*Fig. S1.* Spectroradiometer analysis of the three light conditions used in our experiments.

*Fig. S2.* Validation of the use of absorbance at 550 nm (OD 550 nm) to estimate the cell abundance of *Ostreococcus* cultures.

*Fig. S3*. Examples of cell cycle-related genes upregulated under blue light.

*Fig. S4.* Effect of light quality on the expression of Calvin cycle-related genes.

*Fig. S5.* Effect of light quality on the expression of glycolysis and starch synthesis-related genes.

*Fig. S6*. Effect of light quality on the expression of fatty acid synthesis-related genes.

*Fig. S7*. Effect of light quality on the expression of TCA cycle-related genes.

*Fig. S8.* Effect of light quality on the expression of terpenoid biosynthesis-related genes.

*Fig. S9.* Effect of light quality on the expression of porphyrin and chlorophyll metabolism-related genes.

*Fig. S10.* Effect of light quality on the expression of carotenoid biosynthesis-related genes.

*Fig. S11.* Effect of light quality on the expression of *Ostreococcus* RCC809 genes encoding photoreceptors.

*Fig. S12*. Examples of HPLC chromatograms illustrating the range of pigments detected in the (A) RCC809 and (B) OTTH0595 ecotypes of *Ostreococcus*.

*Table S1.* Lists of differentially expressed genes identified for RCC809 in pairwise comparisons between red and green, red and blue, or blue and green light conditions.

*Table S2.* Light quality-responsive genes related to the cell cycle.

*Table S3.* Light quality-responsive genes with roles in photosystem II assembly.

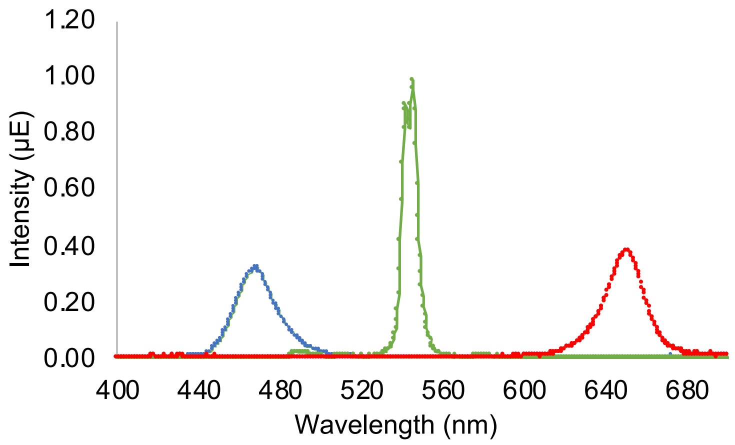


**Fig. S1.** Spectroradiometer analysis of the three light conditions used in our experiments. Peak wavelengths were 650.5 nm for red light, 545 nm for green light, and 467.5 nm for blue light. Total light intensity was 4 μmol photons $m^{-2}$ $s^{-1}$ (4 μE) under all conditions, as indicated by the area under the curves.

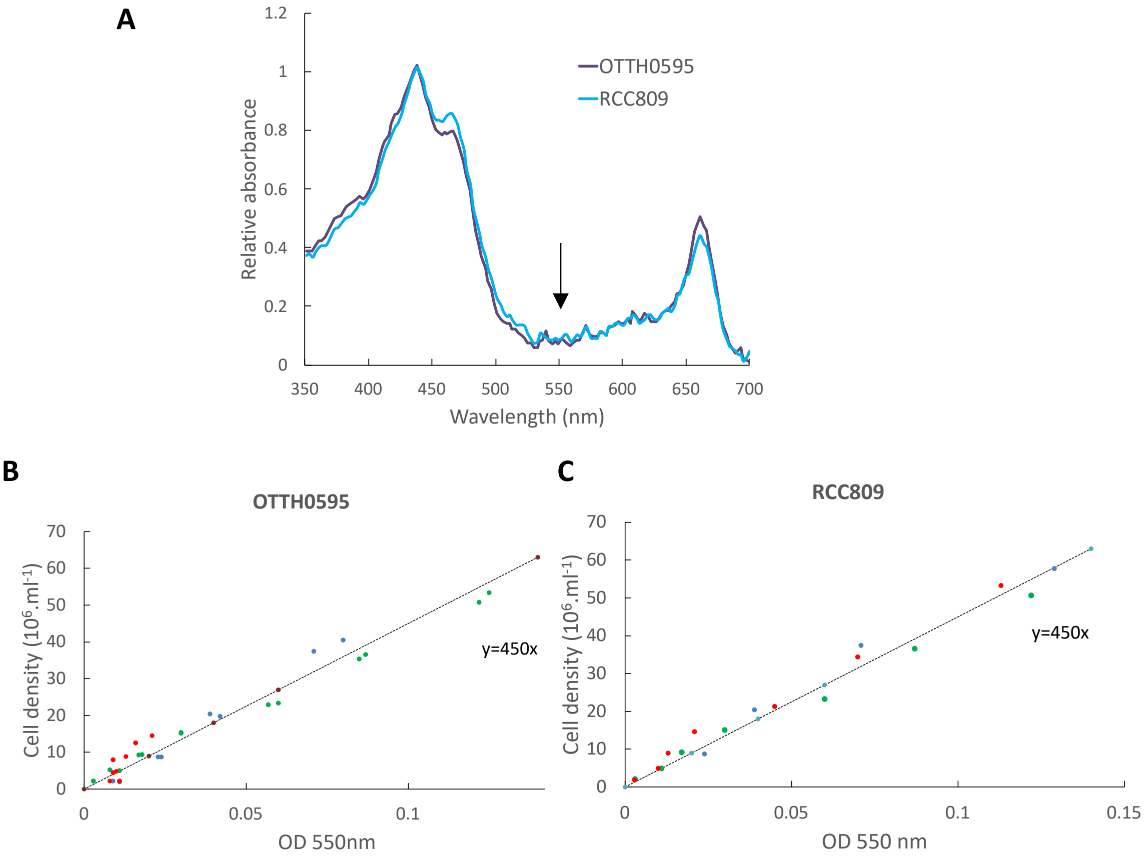


**Fig. S2** Validation of the use of absorbance at 550 nm (OD 550 nm) to estimate the cell abundance of *Ostreococcus* cultures. **(A)** Absorbance spectra of OTTH0595 and RCC809 cultures acclimated to blue light. Absorbance levels shown were calculated relative to 440 nm. The vertical arrow indicates the wavelength (550 nm) used to monitor growth of the cultures. **(B)** and **(C)** illustrate the linear relationship between OD 550 nm and cell density, determined by flow cytometry. The colour of individual data points indicates the relevant growth condition (i. e., red, green or blue light).

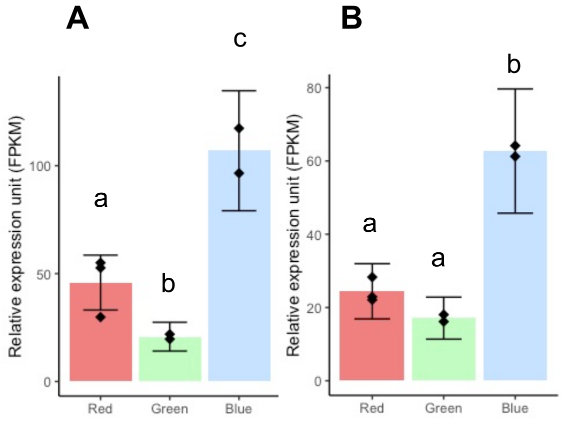


**Fig. S3.** Examples of cell cycle-related genes upregulated under blue light. **(A)** Cyclin-dependent kinase Od14g01080. **(B)** Rad51 DNA recombinase Od08g1730. Different letters indicate significant differences ($p<0.05$) between light conditions, as determined by Student's t-tests. See also Table S2.

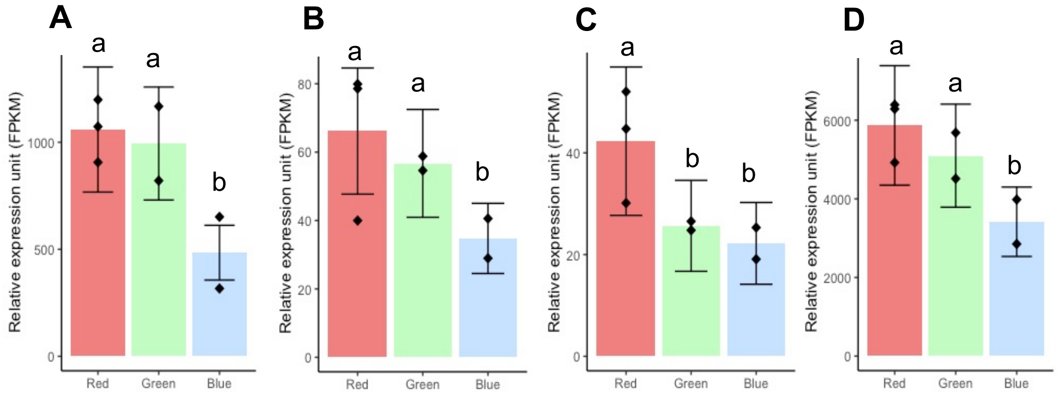


**Fig. S4.** Effect of light quality on the expression of Calvin cycle-related genes. **(A-C)** RuBisCO small subunit genes Od17g01990, Od17g02000 and Od17g02010, respectively. **(D)** RuBisCO activase Od04g02820. Different letters indicate significant differences ($p<0.05$) between light conditions, as determined by Student's t-tests.

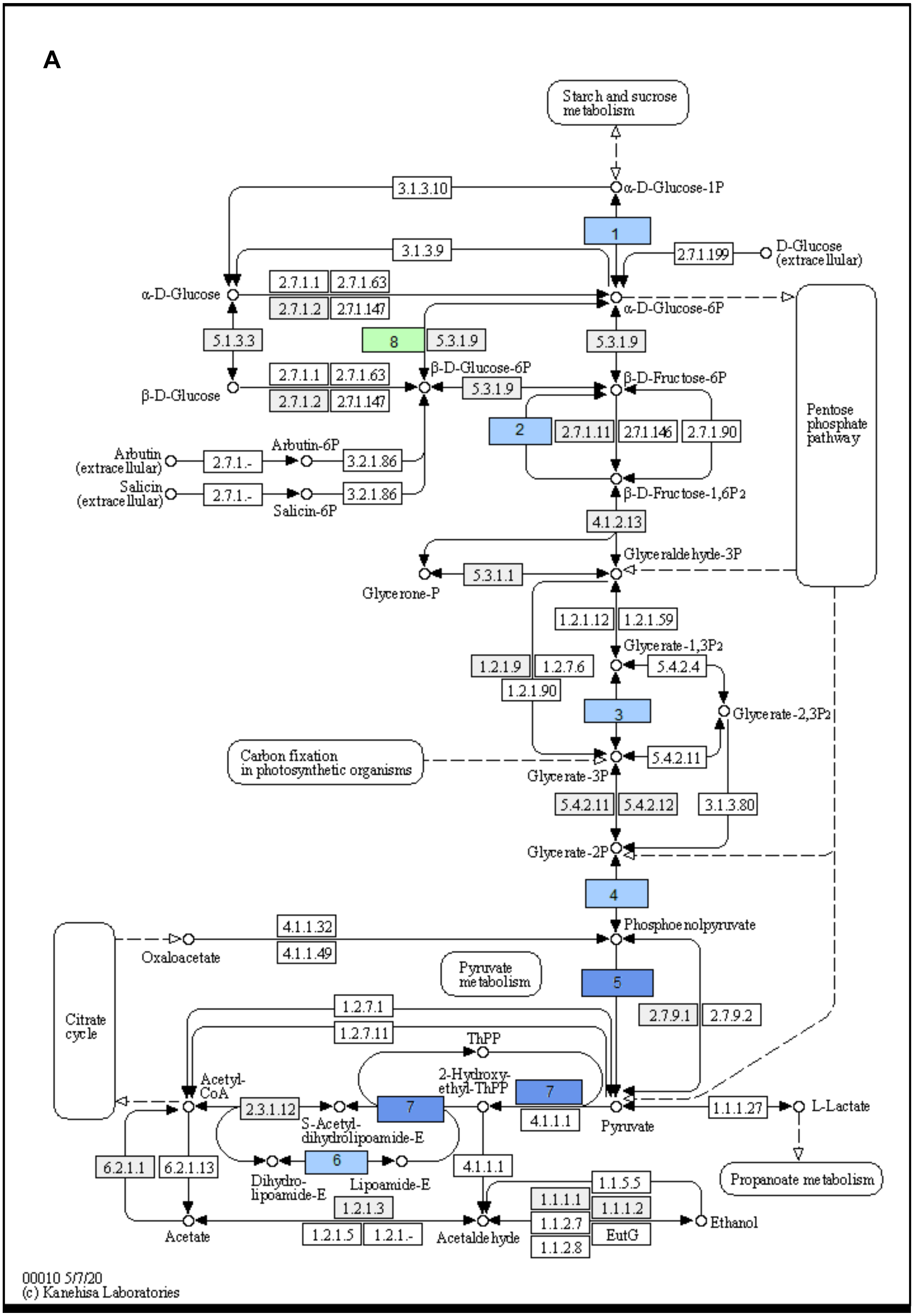
A
Starch and sucrose metabolism
α-D-Glucose-1P
3.1.3.10
1
3.1.3.9
2.7.1.199
D-Glucose (extracellular)
α-D-Glucose
2.7.1.1
2.7.1.63
2.7.1.2
2.7.1.147
α-D-Glucose-6P
5.1.3.3
8
5.3.1.9
5.3.1.9
β-D-Glucose
β-D-Glucose-6P
5.3.1.9
β-D-Fructose-6P
2
2.7.1.11
2.7.1.146
2.7.1.90
Pentose phosphate pathway
Arbutin (extracellular)
2.7.1.-
Arbutin-6P
3.2.1.86
Salicin (extracellular)
2.7.1.-
Salicin-6P
3.2.1.86
β-D-Fructose-1,6P2
4.1.2.13
Glyceraldehyde-3P
Glycerone-P
5.3.1.1
1.2.1.12
1.2.1.59
Glycerate-1,3P2
1.2.1.9
1.2.7.6
1.2.1.90
5.4.2.4
3
Glycerate-2,3P2
Carbon fixation in photosynthetic organisms
5.4.2.11
Glycerate-3P
5.4.2.11
5.4.2.12
3.1.3.80
Glycerate-2P
4
Citrate cycle
Oxaloacetate
4.1.1.32
4.1.1.49
Phosphoenolpyruvate
Pyruvate metabolism
5
1.2.7.1
1.2.7.11
2.7.9.1
2.7.9.2
ThPP
2-Hydroxy-ethyl-ThPP
7
Acetyl-CoA
2.3.1.12
7
4.1.1.1
Pyruvate
1.1.1.27
L-Lactate
S-Acetyl-dihydrolipoamide-E
6
Dihydro-lipoamide-E
Lipoamide-E
6.2.1.1
6.2.1.13
4.1.1.1
Propanoate metabolism
1.1.5.5
1.2.1.3
1.1.1.1
1.1.1.2
Acetate
1.2.1.5
1.2.1.-
Acetaldehyde
1.1.2.7
EutG
Ethanol
1.1.2.8
00010 5/7/20
(c) Kanehisa Laboratories

**B**

| Label | Gene | Enzyme code | Response | Function |
|---|---|---|---|---|
| 1 | Od14g02940 | ec:5.4.2.2 | Up red, down blue | Phosphoglucomutase |
| 2 | Od03g02850 | ec:3.1.3.11 | Down blue | Fructose-bisphosphatase |
| 2 | Od20g01100 | ec:3.1.3.11 | Down blue | Fructose-bisphosphatase |
| 2 | Od09g06250 | ec:3.1.3.11 | Down blue | Fructose-bisphosphatase |
| 2 | Od06g06980 | ec:3.1.3.11 | Down blue | Fructose-bisphosphatase, glyceraldehyde-3P => ribulose-5P |
| 3 | Od06g00660 | ec:2.7.2.3 | Down blue | Phosphoglycerate kinase |
| 4 | Od01g05480 | ec:4.2.1.11 | Down blue | Phosphopyruvate hydratase; Ubiquitinyl hydrolase 1 |
| 5 | Od14g00300 | ec:2.7.1.40 | Down blue | Pyruvate kinase |
| 6 | Od04g00660 | ec:1.8.1.4 | Down blue | Dihydrolipoyl dehydrogenase |
| 7 | Od01g01190 | ec:1.2.4.1 | Up blue | Pyruvate dehydrogenase |
| 5 | Od14g00390 | ec:2.7.1.40 | Up blue | Pyruvate kinase |
| 8 | Od01g01440 | ec:5.1.3.15 | Down green | Glucose-6-phosphate 1-epimerase |

**Fig. S5.** Effect of light quality on the expression of glycolysis and starch synthesis-related genes. **(A)** Annotated KEGG pathway diagram. Steps of the pathway where expression of enzymes is affected by light quality are indicated by numbered, colour-coded labels. Colours indicate the wavelengths that induce the response. Darker colours indicate induction, lighter colours indicate repression. **(B)** Corresponding genes and their light quality responses.

**A**

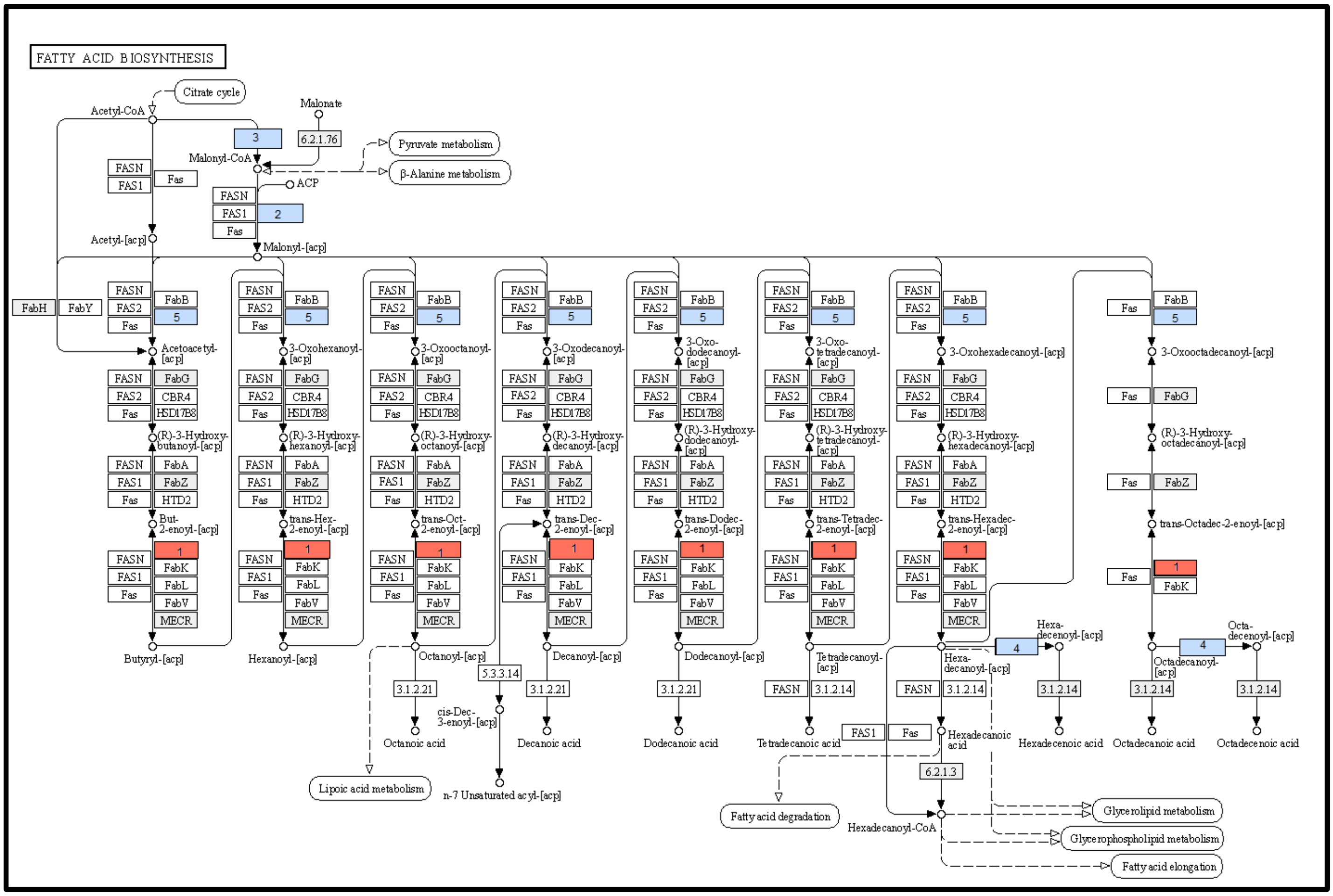


**B**

| Label | Gene | Enzyme code | Response | Function |
|---|---|---|---|---|
| 1 | Od07g04250 | ec:1.3.1.9 | Up red | Fatty-acid synthase; Enoyl-[acyl-carrier-protein] reductase (NADH); FabI |
| 2 | Od09g03480 | ec:2.3.1.39 | Down blue | Fatty-acid synthase; [Acyl-carrier-protein] S-malonyltransferase; FabD |
| 3 | Od01g03440 | ec:6.4.1.2 | Down blue | Acetyl-CoA carboxylase |
| 4 | Od04g01890 | ec:1.14.19.2 | Down blue | Stearoyl-[acyl-carrier-protein] 9-desaturase |
| 5 | Od14g01920 | ec:2.3.1.179 | Down blue | Fatty-acid synthase; Beta-ketoacyl-[acyl-carrier-protein] synthase I; FabF |

**Fig. S6.** Effect of light quality on the expression of fatty acid synthesis-related genes. **(A)** Annotated KEGG pathway diagram. **(B)** Corresponding genes and their light quality responses.

A

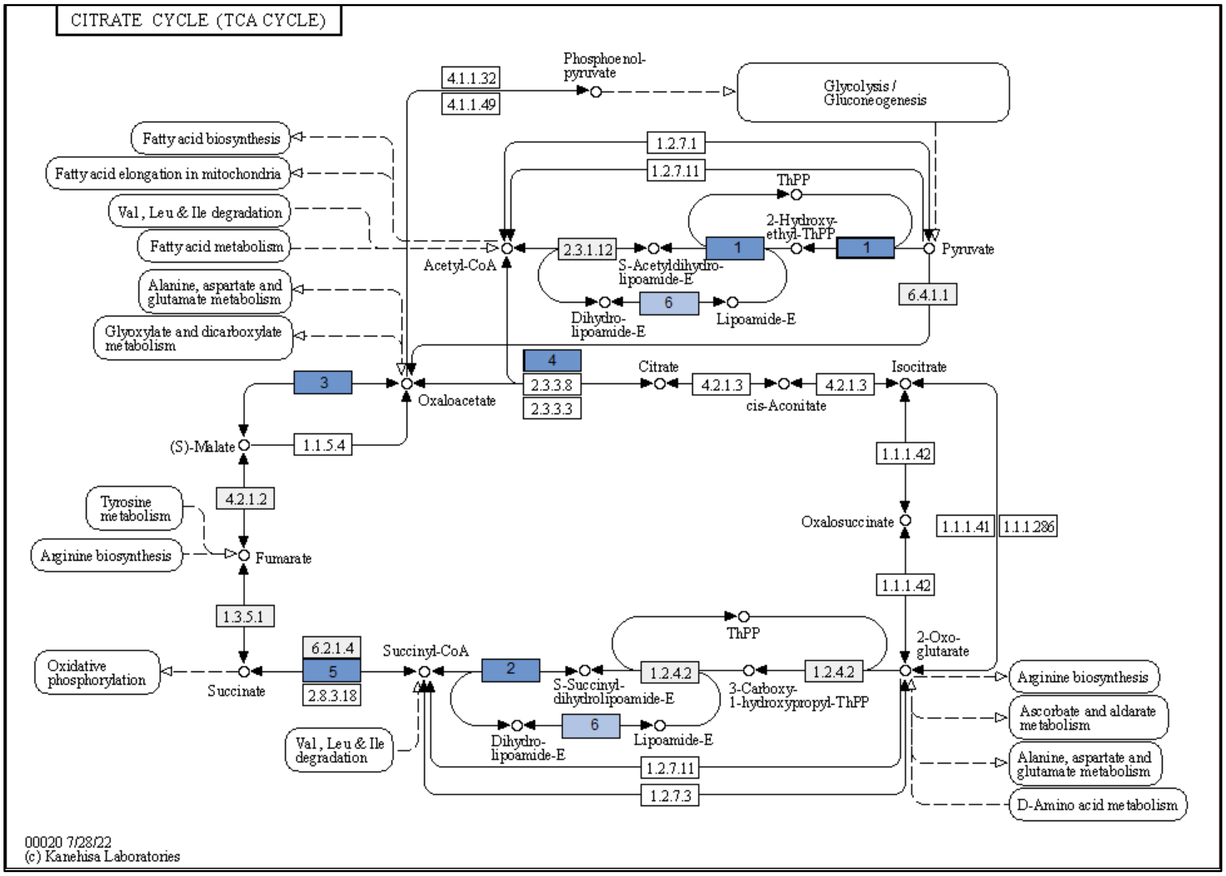


B

| Label | Gene | Enzyme code | Response | Function |
|---|---|---|---|---|
| 1 | Od01g01190 | ec:1.2.4.1 | Up blue | Pyruvate dehydrogenase, glycolysis |
| 2 | Od02g03200 | ec:2.3.1.61 | Up blue | Succinyltransferase, amino acid metabolism |
| 3 | Od03g03160 | ec:1.1.1.37 | Up blue, down green | Malate dehydrogenase, cysteine and methionine metabolism |
| 3 | Od06g02230 | ec:1.1.1.37 | Up blue | Malate dehydrogenase, cysteine and methionine metabolism |
| 3 | Od08g00070 | ec:1.1.1.37 | Up blue | Malate dehydrogenase |
| 4 | Od05g01850 | ec:2.3.3.1 | Up blue | Citrate (Si)-synthase |
| 5 | Od06g07420 | ec:6.2.1.5 | Up blue | Succinate--CoA ligase (ADP-forming) |
| 6 | Od04g00660 | ec:1.8.1.4 | Down blue | Dihydrolipoyl dehydrogenase, glycolysis |

**Fig. S7.** Effect of light quality on the expression of TCA cycle-related genes. **(A)** Annotated KEGG pathway diagram. **(B)** Corresponding genes and their light quality responses.

A

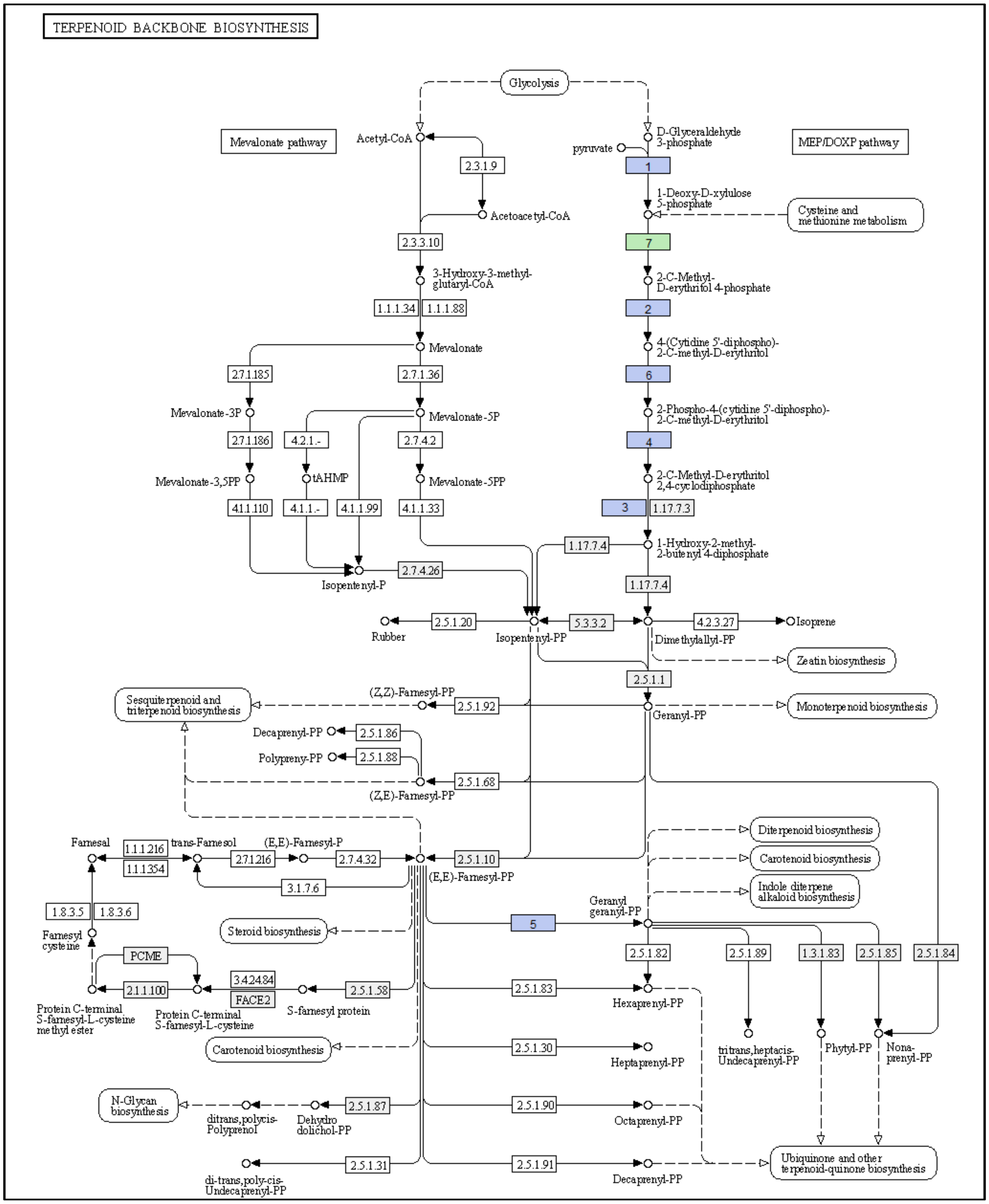

TERPENOID BACKBONE BIOSYNTHESIS
Glycolysis
Mevalonate pathway
MEP/DOXP pathway
Acetyl-CoA
2.3.1.9
Acetoacetyl-CoA
2.3.3.10
3-Hydroxy-3-methyl-glutaryl-CoA
1.1.1.34
1.1.1.88
Mevalonate
2.7.1.185
2.7.1.36
Mevalonate-3P
Mevalonate-5P
2.7.1.186
4.2.1.-
2.7.4.2
Mevalonate-3,5PP
tAHMP
Mevalonate-5PP
4.1.1.110
4.1.1.-
4.1.1.99
4.1.1.33
Isopentenyl-P
2.7.4.26
D-Glyceraldehyde 3-phosphate
pyruvate
1
1-Deoxy-D-xylulose 5-phosphate
Cysteine and methionine metabolism
7
2-C-Methyl-D-erythritol 4-phosphate
2
4-(Cytidine 5'-diphospho)-2-C-methyl-D-erythritol
6
2-Phospho-4-(cytidine 5'-diphospho)-2-C-methyl-D-erythritol
4
2-C-Methyl-D-erythritol 2,4-cyclodiphosphate
3
1.17.7.3
1.17.7.4
1-Hydroxy-2-methyl-2-butenyl 4-diphosphate
1.17.7.4
Rubber
2.5.1.20
Isopentenyl-PP
5.3.3.2
Dimethylallyl-PP
4.2.3.27
Isoprene
Zeatin biosynthesis
2.5.1.1
Sesquiterpenoid and triterpenoid biosynthesis
(Z,Z)-Farnesyl-PP
2.5.1.92
Geranyl-PP
Monoterpenoid biosynthesis
Decaprenyl-PP
2.5.1.86
Polypreny-PP
2.5.1.88
2.5.1.68
(Z,E)-Farnesyl-PP
Farnesal
1.1.1.216
1.1.1.354
trans-Farnesol
2.7.1.216
(E,E)-Farnesyl-P
2.7.4.32
2.5.1.10
(E,E)-Farnesyl-PP
3.1.7.6
Diterpenoid biosynthesis
Carotenoid biosynthesis
Indole diterpene alkaloid biosynthesis
1.8.3.5
1.8.3.6
Farnesyl cysteine
Steroid biosynthesis
5
Geranyl geranyl-PP
PCME
3.4.24.84
FACE2
2.1.1.100
2.5.1.58
S-farnesyl protein
Protein C-terminal S-farnesyl-L-cysteine methyl ester
Protein C-terminal S-farnesyl-L-cysteine
2.5.1.82
2.5.1.89
1.3.1.83
2.5.1.85
2.5.1.84
2.5.1.83
Hexaprenyl-PP
tritrans,heptacis-Undecaprenyl-PP
Phytyl-PP
Nona-prenyl-PP
Carotenoid biosynthesis
2.5.1.30
Heptaprenyl-PP
N-Glycan biosynthesis
ditrans,polycis-Polyprenol
Dehydro dolichol-PP
2.5.1.87
2.5.1.90
Octaprenyl-PP
Ubiquinone and other terpenoid-quinone biosynthesis
di-trans,poly-cis-Undecaprenyl-PP
2.5.1.31
2.5.1.91
Decaprenyl-PP

**B**

| Label | Gene | Enzyme code | Response | Function |
|---|---|---|---|---|
| 1 | Od02g01640 | ec:2.2.1.7 | Down blue | 1-deoxy-D-xylulose-5-phosphate synthase |
| 2 | Od07g03920 | ec:2.7.7.60 | Down blue | 2-C-methyl-D-erythritol 4-phosphate cytidylyltransferase |
| 3 | Od08g01130 | ec:1.17.7.1 | Down blue | Ferredoxin |
| 3 | Od09g05360 | ec:1.17.7.1 | Down blue | Ferredoxin |
| 4 | Od11g00960 | ec:4.6.1.12 | Down blue | 2-C-methyl-D-erythritol 2,4-cyclodiphosphate synthase |
| 5 | Od12g01950 | ec:2.5.1.29 | Down blue | Geranylgeranyl diphosphate synthase |
| 6 | Od15g01050 | ec:2.7.1.148 | Down blue | 4-(cytidine 5'-diphospho)-2-C-methyl-D-erythritol kinase |
| 7 | Od04g02130 | ec:1.1.1.267 | Down green | 1-deoxy-D-xylulose-5-phosphate reductoisomerase |

**Fig. S8.** Effect of light quality on the expression of terpenoid biosynthesis-related genes. **(A)** Annotated KEGG pathway diagram. **(B)** Corresponding genes and their light quality responses.

A

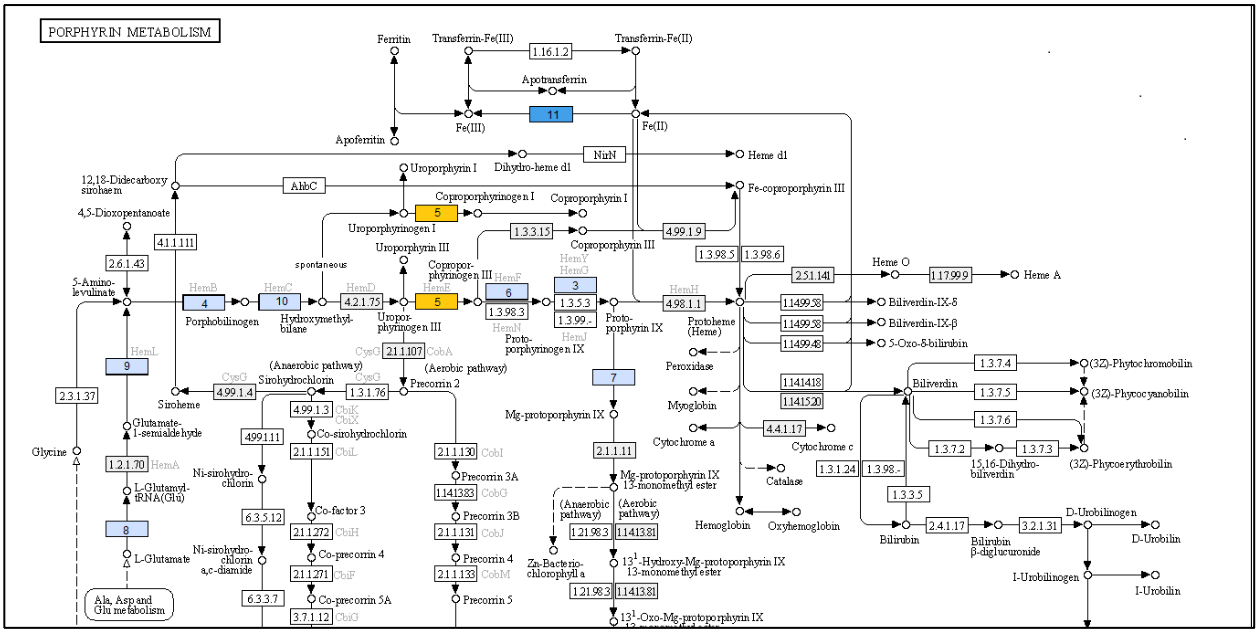
PORPHYRIN METABOLISM
Ferritin
Transferrin-Fe(III)
1.16.1.2
Transferrin-Fe(II)
Apotransferrin
11
Fe(III)
Fe(II)
Apoferritin
NirN
Heme d1
Dihydro-heme d1
Uroporphyrin I
12,18-Didecarboxy sirohaem
AhbC
Fe-coproporphyrin III
4,5-Dioxopentanoate
Coproporphyrinogen I
Coproporphyrin I
5
4.1.1.111
Uroporphyrinogen I
1.3.3.15
4.99.1.9
2.6.1.43
Uroporphyrin III
Coproporphyrin III
spontaneous
1.3.98.5
1.3.98.6
5-Amino-levulinate
HemB
4
HemC
10
HemD
4.2.1.75
HemE
HemF
6
HemY
HemG
3
HemH
4.98.1.1
2.5.1.141
Heme O
1.17.99.9
Heme A
Porphobilinogen
Hydroxymethyl-bilane
Uropor-phyrinogen III
Copropor-phyrinogen III
1.3.98.3
1.3.5.3
1.3.99.-
Proto-porphyrin IX
Protoheme (Heme)
1.14.99.58
Biliverdin-IX-δ
Biliverdin-IX-β
1.14.99.48
5-Oxo-δ-bilirubin
HemN
Proto-porphyrinogen IX
HemJ
HemL
9
CysG
2.1.1.107
CobA
(Anaerobic pathway)
(Aerobic pathway)
Peroxidase
1.3.7.4
(3Z)-Phytochromobilin
Sirohydrochlorin
7
1.14.14.18
1.14.15.20
Biliverdin
1.3.7.5
(3Z)-Phycocyanobilin
2.3.1.37
Siroheme
4.99.1.4
1.3.1.76
Precorrin 2
Myoglobin
1.3.7.6
Mg-protoporphyrin IX
4.99.1.3
CbiK
CbiX
Glutamate-1-semialdehyde
4.99.1.11
Co-sirohydrochlorin
Cytochrome a
4.4.1.17
Cytochrome c
1.3.7.2
1.3.7.3
Glycine
2.1.1.151
CbiL
2.1.1.130
CobI
2.1.1.11
15,16-Dihydro-biliverdin
(3Z)-Phycoerythrobilin
1.2.1.70
HemA
1.3.1.24
1.3.98.-
Ni-sirohydro-chlorin
Precorrin 3A
Mg-protoporphyrin IX 13-monomethyl ester
Catalase
L-Glutamyl-tRNA(Glu)
1.14.13.83
CobG
1.3.3.5
Co-factor 3
Precorrin 3B
(Anaerobic pathway)
(Aerobic pathway)
Hemoglobin
Oxyhemoglobin
D-Urobilinogen
6.3.5.12
2.1.1.272
CbiH
2.1.1.131
CobJ
1.21.98.3
1.14.13.81
Bilirubin
2.4.1.17
3.2.1.31
8
D-Urobilin
Bilirubin β-diglucuronide
L-Glutamate
Ni-sirohydro-chlorin a,c-diamide
Co-precorrin 4
Precorrin 4
Zn-Bacterio-chlorophyll a
2.1.1.271
CbiF
2.1.1.133
CobM
I-Urobilinogen
I-Urobilin
Ala, Asp and Glu metabolism
6.3.3.7
Co-precorrin 5A
Precorrin 5
3.7.1.12
CbiG

B

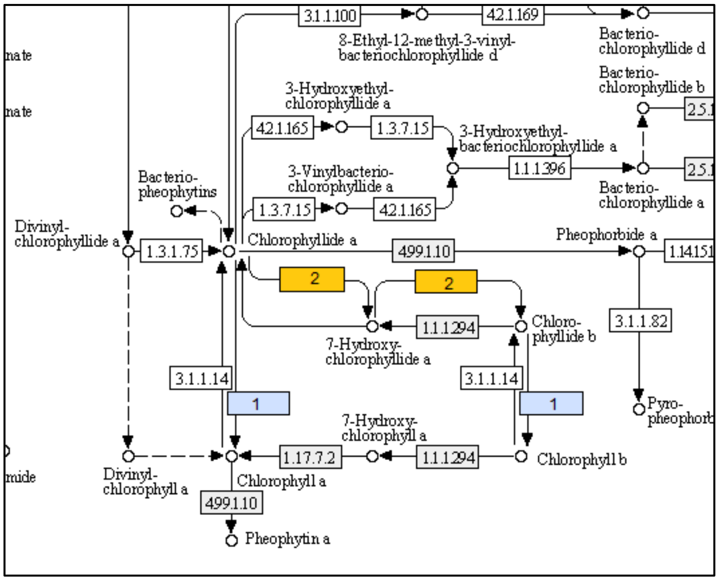
3.1.1.100
4.2.1.169
8-Ethyl-12-methyl-3-vinyl-bacteriochlorophyllide d
Bacterio-chlorophyllide d
Bacterio-chlorophyllide b
3-Hydroxyethyl-chlorophyllide a
4.2.1.165
1.3.7.15
3-Hydroxyethyl-bacteriochlorophyllide a
1.1.1.396
Bacterio-chlorophyllide a
3-Vinylbacterio-chlorophyllide a
1.3.7.15
4.2.1.165
Bacterio-pheophytins
Divinyl-chlorophyllide a
1.3.1.75
Chlorophyllide a
4.99.1.10
Pheophorbide a
2
2
7-Hydroxy-chlorophyllide a
1.1.1.294
Chloro-phyllide b
3.1.1.82
3.1.1.14
1
3.1.1.14
1
Pyro-
7-Hydroxy-chlorophyll a
Divinyl-chlorophyll a
1.17.7.2
Chlorophyll a
1.1.1.294
Chlorophyll b
4.99.1.10
Pheophytin a

C

| Label | Gene | Enzyme code | Response | Function |
|---|---|---|---|---|
| 1 | Od08g01040 | ec:2.5.1.62 | Down blue | Chlorophyll synthase |
| 2 | Od09g01000 | ec:1.14.13.122 | Down blue | Chlorophyllide a oxygenase |
| 3 | Od06g03440 | ec:1.3.3.4 | Down blue | Protoporphyrinogen oxidase |
| 4 | Od01g04650 | ec:4.2.1.24 | Down blue | Porphobilinogen synthase |
| 5 | Od12g03120 | ec:4.1.1.37 | Down blue | Uroporphyrinogen decarboxylase |
| 5 | Od16g02600 | ec:4.1.1.37 | Down blue | Uroporphyrinogen decarboxylase |
| 6 | Od06g04640 | ec:1.3.3.3 | Down blue | Coproporphyrinogen oxidase |
| 7 | Od01g01020 | ec:6.6.1.1 | Down blue | Magnesium chelatase |
| 7 | Od05g05120 | ec:6.6.1.1 | Down blue | Magnesium chelatase |
| 7 | Od04g03220 | ec:6.6.1.1 | Down blue | Magnesium chelatase |
| 7 | Od02g02530 | ec:6.6.1.1 | Down blue | Magnesium chelatase |
| 8 | Od06g03340 | ec:6.1.1.17 | Down blue | Glutamate--tRNA ligase |
| 9 | Od19g01200 | ec:5.4.3.8 | Down blue | Glutamate-1-semialdehyde aminotransferase |
| 10 | Od05g01350 | ec:2.5.1.61 | Down blue | Porphobilinogen deaminase |
| 11 | Od02g03670 | ec:1.16.3.1 | Up blue | Ferroxidase, ferritin-like superfamily |
| 5 | Od12g03130 | ec:4.1.1.37 | Up blue | Uroporphyrinogen decarboxylase |
| 2 | Od14g00700 | ec:1.14.13.122 | Up blue | Chlorophyllide a oxygenase |
| 5 | Od05g01200 | ec:4.1.1.37 | Down green | Uroporphyrinogen decarboxylase |

**Fig. S9.** Effect of light quality on the expression of porphyrin and chlorophyll metabolism-related genes. **(A, B)** Annotated KEGG pathway diagram for porphyrin and chlorophyll metabolism, respectively. Steps highlighted in orange are those for which light quality responses were inconsistent, i. e. some enzyme isoforms were up-regulated while others were down-regulated in the same condition **(C)** Corresponding genes and their light quality responses.

**A**

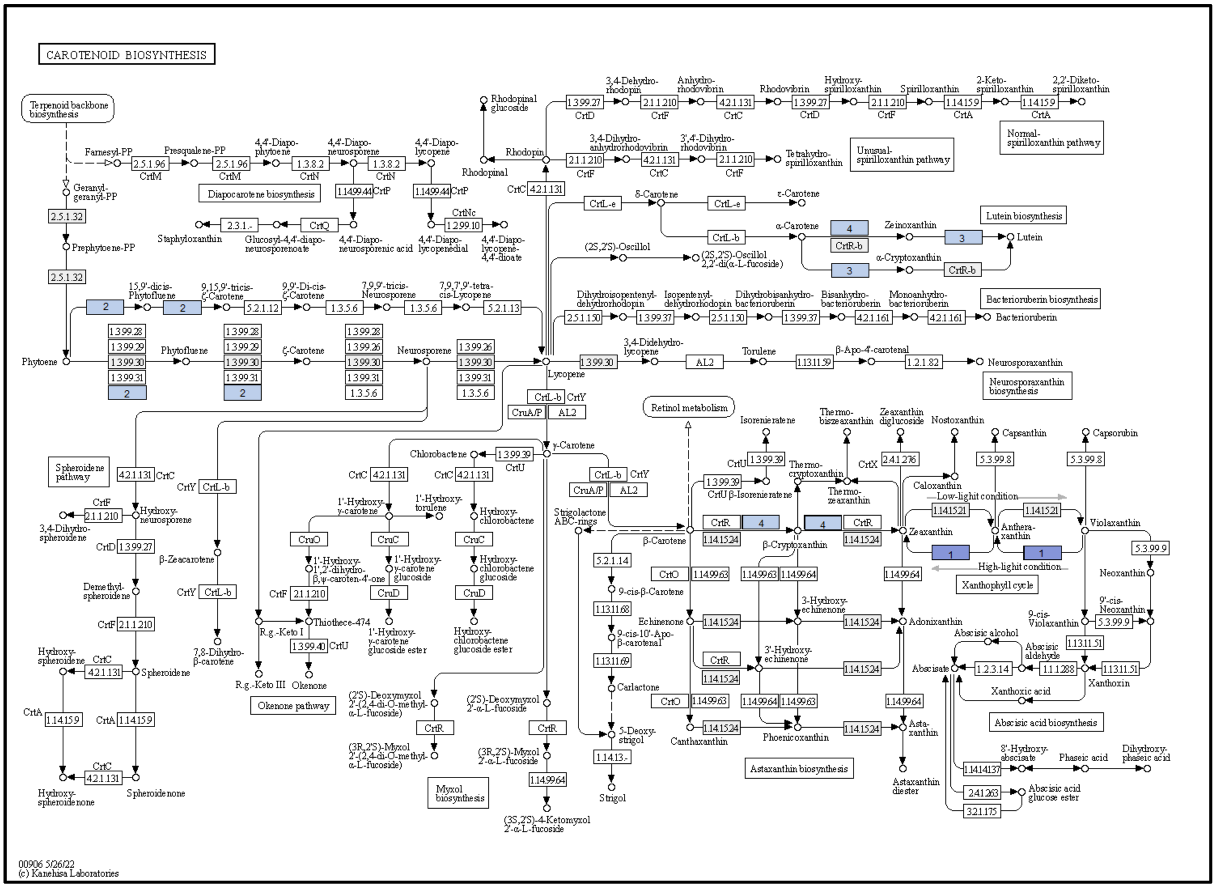


**B**

| Label | Gene | Enzyme code | Response | Function |
|---|---|---|---|---|
| 1 | Od11g00170 | ec:1.23.5.1 | Up blue | Violaxanthin de-epoxidase |
| 2 | Od01g00740 | ec:1.3.5.5 | Up red | Phytoene desaturase |
| 2 | Od09g00820 | ec:1.3.5.5 | Down blue | Phytoene desaturase |
| 3 | Od09g03950 | ec:1.14.14.158 | Down blue | Cytochrome P450, LUT1, CYP97C1 |
| 4 | Od13g00750 | LUT5 | Down blue | Cytochrome P450 |

**Fig. S10.** Effect of light quality on the expression of carotenoid biosynthesis-related genes. **(A)** Annotated KEGG pathway diagram. Enzymes whose expression is affected by light quality are

indicated by numbers on coloured labels. **(B)** Corresponding genes and their light quality responses.

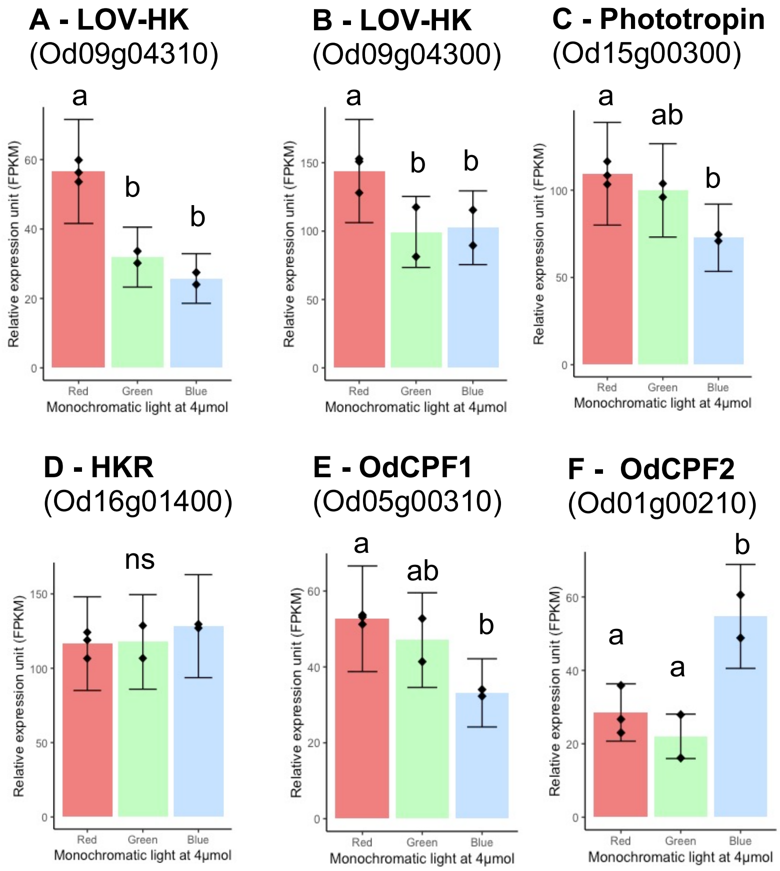


**Fig. S11.** The effect of light quality on the expression of *Ostreococcus* RCC809 genes encoding photoreceptors. Different letters indicate significant differences, and ns indicates no significant differences.

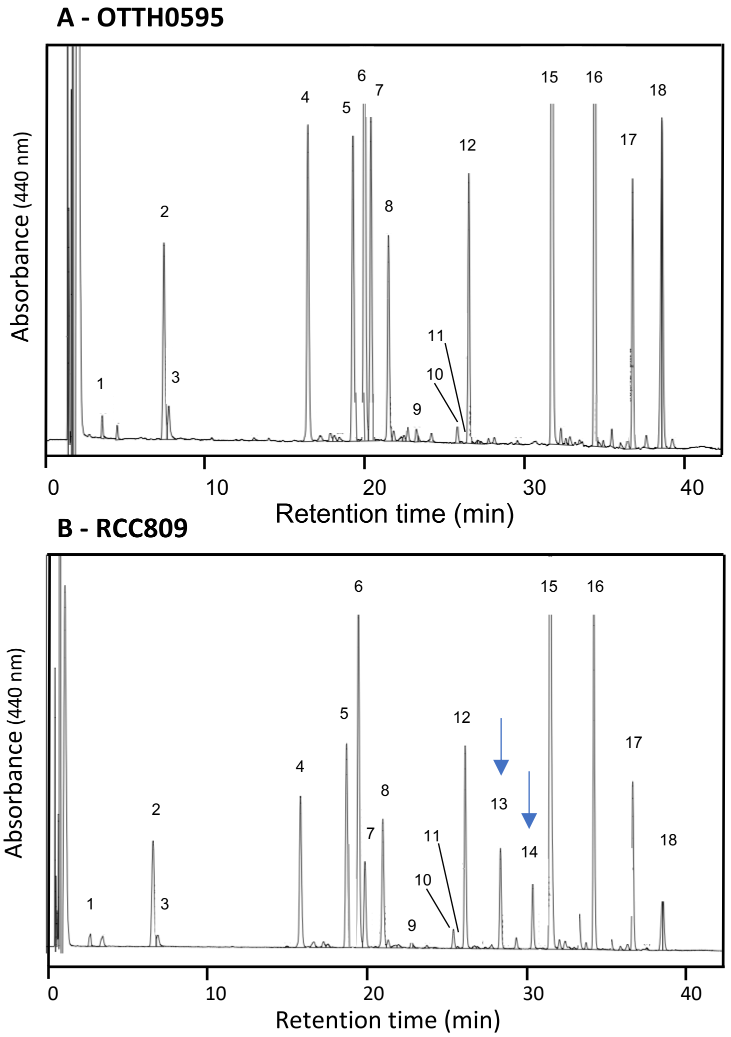


| Peak number | Pigment | Retention time (min) | Peak number | Pigment | Retention time (min) |
|---|---|---|---|---|---|
| 1 | chlorophyllide b | 3.5 | 10 | zeaxanthin | 25.8 |
| 2 | MgDVP | 7.4 | 11 | lutein | 26.1 |
| 3 | chlorophyllide a | 7.7 | 12 | dihydrolutein | 31.8 |
| 4 | uriolide | 16.4 | 13 | chlorophyll b-like | 28.7 |
| 5 | neoxanthin | 19.3 | 14 | chlorophyll b-like | 30.7 |
| 6 | prasinoxanthin | 20.0 | 15 | chlorophyll b | 31.7 |
| 7 | violaxanthin | 20.4 | 16 | chlorophyll a | 34.5 |
| 8 | micromonal | 21.5 | 17 | unknown carotenoid | 36.9 |
| 9 | antheraxanthin | 23.4 | 18 | β-carotene | 38.7 |

**Fig. S12** Examples of HPLC chromatograms illustrating the diversity of pigments present in the **(A)** OTTH95 and **(B)** RCC809 ecotypes of *Ostreococcus*. Two chl*b* like pigments found in RCC809 but not in OTTH0595 are highlighted with blue arrows.

**Table S1** Lists of differentially expressed genes identified in pairwise comparisons between red and green, red and blue, or blue and green light conditions (provided as a separate Excel file).

**Table S2** Light quality-responsive genes related to the cell cycle.

| Gene | Light response | Description |
|---|---|---|
| Od01g01520 | Down in blue | Single-stranded DNA-binding protein, DNA replication |
| Od05g01780 | Down in blue | DNA replication licensing factor, protein ubiquitination |
| Od07g01730 | Down in blue | Lysine-specific demethylase 8, protein binding |
| Od07g02050 | Down in blue | Predicted, GTPase activity, chloroplast fission, cell division |
| Od07g04740 | Down in blue | Cell division FtsY homolog, chloroplastic, GTP binding |
| Od10g01360 | Down in blue | Resistance-nodulation-cell division superfamily |
| Od01g04460 | Down in green | DNA replication licensing factor Mcm6, helicase |
| Od02g01320 | Down in green | DNA replication licensing factor Mcm7 helicase |
| Od02g01330 | Down in green | DNA replication licensing factor Mcm7 helicase |
| Od02g02310 | Down in green | DNA replication licensing factor Mcm3 helicase |
| Od05g00150 | Down in green | Replication factor C subunit 3 DNA replication |
| Od10g01480 | Down in green | ATP-dependent helicase/nuclease DNA2 isoform X1, 5'-flap DNA replication, Okazaki fragment processing |
| Od17g03020 | Down in green | P4 family, helicase activity, ATP binding, DNA replication |
| Od01g02860 | Up in blue | Histone H3, nucleosomal DNA binding, meiotic chromosome separation |
| Od02g03320 | Up in blue | Predicted, peptide dephosphorylation, positive regulation of cell cycle, G2/M phase transition |
| Od02g03690 | Up in blue | Serine/threonine-protein kinase 24, regulation of cell cycle |
| Od02g03890 | Up in blue | Condensin complex component, non-smc subunit, mitotic chromosome condensation, cell division |
| Od03g01380 | Up in blue | Predicted, replication, recombination, 3'-5' helicase activity |
| Od04g04750 | Up in blue | Group J -like protein, DNA helicase, replication fork |
| Od06g00290 | Up in blue | Predicted protein, binding, cell division |
| Od06g04320 | Up in blue | Predicted protein, DNA binding, replication |

| | | |
|---|---|---|
| Od08g00520 | Up in blue | DNA replication, cell death, regulation of cell cycle |
| Od08g01730 | Up in blue | RAD51 homolog, regulation of transcription, double-strand break repair, recombination-dependent replication fork |
| Od09g01440 | Up in blue | Predicted protein, mitotic cell cycle, centromeric region |
| Od09g03260 | Up in blue | Predicted protein, regulation of cell cycle |
| Od12g01680 | Up in blue | Probable DNA helicase MCM8, DNA replication initiation |
| Od12g02990 | Up in blue | Ribonuclease HII, RNA-DNA hybrid activity, mismatch repair, removal of RNA primer |
| Od20g01820 | Up in blue | Predicted, heterochromatin assembly involved in chromatin silencing, mitotic DNA replication |
| Od04g00830 | Up in blue | Predicted, DNA replication initiation |
| Od04g04360 | Up in blue / Down in green | Predicted, DNA replication initiation, cell division |
| Od06g02850 | Up in blue / Down in green | Leading strand elongation, mismatch repair, DNA pol |
| Od07g02890 | Up in blue / Down in green | Predicted, anaphase-promoting complex, regulation of mitotic metaphase/anaphase transition, cell division |
| Od08g00650 | Up in blue / Down in green | DNA pol catalytic subunit, synthesis of RNA primer; DNA replication initiation, lagging strand elongation, transcription |
| Od11g01890 | Up in blue / Down in green | Probable DNA primase large subunit, DNA replication, transcription |
| Od11g02480 | Up in blue / Down in green | DNA polymerase alpha subunit B, DNA replication |
| Od12g02680 | Up in blue / Down in green | DNA topoisomerase 1 beta, DNA replication, chromatin remodelling, chromosome segregation, replication fork |
| Od13g01180 | Up in blue / Down in green | DNA primase small subunit isoform X2, DNA replication, transcription, DNA-templated |
| Od14g01080 | Up in blue / Down in green | Predicted, G1/S mitotic cell cycle, cyclin-dependent kinase activity, regulation of G2/M mitotic cell cycle |
| Od17g01560 | Up in blue / Down in green | Replication protein A 70 kDa DNA-binding subunit B, DNA repair; DNA recombination |
| Od20g01140 | Up in blue / Down in green | Predicted, DNA helicase, DNA replication initiation, MCM complex, cell division |
| Od04g04480 | Up in red | Predicted, histone methyltransferase (H3-K79 specific), regulation of cell cycle |
| Od10g02070 | Up in red | DNA replication, helicase, nucleoside-triphosphatase |

Genes indicated as “Up in blue / Down in green” were identified as up-regulated in blue light relative to red and green light, as well as down-regulated in green light relative to red and blue light.

**Table S3** Light-quality responsive genes with roles in photosystem II assembly.

| Light response | Gene | Description |
|---|---|---|
| Up blue versus red and green | Od04g05310 | Predicted protein, photosystem II assembly |
| Up blue versus red and green | Od07g00100 | Photosystem II 22 kDa protein, chloroplast |
| Up blue versus red and green | Od02g04230 | Putative photosystem II 22 kDa protein, high light induced |
| Up blue versus red | Od06g01970 | Photosystem II S4 domain protein |
| Up blue versus red | Od02g00750 | PsbP domain 7, photosystem II oxygen evolving complex |
| Up blue versus green | Od11g00160 | Photosystem II reaction centre PSB28, 13kD Protein |
| Down blue | Od14g01690 | Predicted, Mog1/PsbP, photosystem II oxygen evolving complex |
